# Feeding Cells Induced by Phytoparasitic Nematodes Require γ-Tubulin Ring Complex for Microtubule Reorganization

**DOI:** 10.1371/journal.ppat.1002343

**Published:** 2011-12-01

**Authors:** Mohamed Youssef Banora, Natalia Rodiuc, Fabien Baldacci-Cresp, Andrei Smertenko, Teresa Bleve-Zacheo, Maria Teresa Mellilo, Mansour Karimi, Pierre Hilson, Jean-Luc Evrard, Bruno Favery, Gilbert Engler, Pierre Abad, Janice de Almeida Engler

**Affiliations:** 1 Unité Mixte de Recherches Interactions Biotiques et Santé Végétale, INRA-CNRS-UNS, Sophia Antipolis, France; 2 School of Biological and Biomedical Sciences, Durham University, Durham, United Kingdom; 3 Istituto di Nematologia Agraria, CNR, Bari, Italia; 4 Department of Plant Systems Biology, Flanders Institute for Biotechnology, Ghent, Belgium; 5 Institut de Biologie Moléculaire des Plantes, CNRS, Strasbourg, France; Virginia Polytechnic Institute and State University, United States of America

## Abstract

Reorganization of the microtubule network is important for the fast isodiametric expansion of giant-feeding cells induced by root-knot nematodes. The efficiency of microtubule reorganization depends on the nucleation of new microtubules, their elongation rate and activity of microtubule severing factors. New microtubules in plants are nucleated by cytoplasmic or microtubule-bound γ-tubulin ring complexes. Here we investigate the requirement of γ-tubulin complexes for giant feeding cells development using the interaction between *Arabidopsis* and *Meloidogyne spp.* as a model system. Immunocytochemical analyses demonstrate that γ-tubulin localizes to both cortical cytoplasm and mitotic microtubule arrays of the giant cells where it can associate with microtubules. The transcripts of two *Arabidopsis* γ-tubulin (*TUBG1* and *TUBG2*) and two γ-tubulin complex proteins genes (*GCP3* and *GCP4*) are upregulated in galls. Electron microscopy demonstrates association of GCP3 and γ-tubulin as part of a complex in the cytoplasm of giant cells. Knockout of either or both γ-tubulin genes results in the gene dose-dependent alteration of the morphology of feeding site and failure of nematode life cycle completion. We conclude that the γ-tubulin complex is essential for the control of microtubular network remodelling in the course of initiation and development of giant-feeding cells, and for the successful reproduction of nematodes in their plant hosts.

## Author Summary

Among plant pathogens, sedentary endoparasitic nematodes are one of the most damaging pests in global agriculture. Nematodes are greatly resistant due to a broad physiological variability, consequently difficult to fight against. The use of pesticides is highly pollutant to the environment and therefore new strategies must be envisaged. As nematodes induce fragmentation and long-term rearrangements of the plant cytoskeleton during infection, manipulation of cytoskeleton components necessary for parasitism could be used as targets for the development of resistant plants provoking the awareness of biotechnology companies and crop breeders in developing new strategies for the control of pathogen infection. Here we report the first stable γ-tubulin-GFP expressing plant line and provide compelling evidence for the physical interaction between components of the γTuRC, γ-tubulin and γ-tubulin-complex protein 3 (GCP3) as part of free cytoplasmic and microtubules associated complexes. We show here that γTuRC is an essential component of the microtubule nucleation machinery during giant cell development. The reduction of γ-tubulin and GCP4 levels compromises γTuRC functioning and affects microtubule nucleation in giant-feeding cells, delaying their development and affecting nematode reproduction. We conclude that upregulation of microtubule nucleation induced by γTuRC is essential for the nematode parasitism and this process can be targeted in order to protect plants against nematode infection.

## Introduction

Root-knot nematodes (RKN) *Meloidogyne spp.* are minuscule worms which are widespread in the soil. They are obligate sedentary phyto-endoparasites known to infect above 3000 plant species. In the course of a compatible interaction, the nematodes of the genera *Meloidogyne* are able to alter the host plant metabolic pathways to their own benefit [1]. The parasitic cycle commences when the motile second-stage juvenile (J2) penetrates a root in the elongation zone [2]. This infective stage nematode migrates via intercellular space of the root cortex towards the root tip and then moves acropetally along a xylem pole to the differentiation zone of the root vascular tissue where it establishes the feeding site by altering the developmental and metabolic program of the vascular parenchymal cells [3]. A typical nematode feeding site (NFS) consists of 6 to 8 hypertrophic cells, named giant cells, with dense cytoplasm containing numerous organelles and characterised by high metabolic activity [4]. These cells serve as the exclusive source of nutrients for the nematode until their reproduction.

In the course of differentiation giant cells undergo karyokinesis followed by incomplete cytokinesis as well as endoreduplication cycles, resulting in the accumulation of multiple enlarged nuclei [5], [6]. This is accompanied by the partial depolymerisation/fragmentation of both main components of plant cytoskeleton: microtubules (MTs) and actin filaments [7]. The reorganization of the cytoskeleton is essential for establishment of the feeding site and successful nematode reproduction [7]–[9].

Microtubules are dynamic filaments formed by polymerization of heterodimeric protein α-/β-tubulin. They are essential for the spatial organization of the cytoplasm, establishment of the cell shape and polarity, cell division, intracellular transport and cell wall deposition. In plants MTs form four functionally specialized arrays: 1) interphase cortical network regulates the cell architecture including the direction of cell expansion; 2) preprophase band (PPB) during G2/M transition predicts site of the forthcoming division [3,10) mitotic spindle separates daughter chromatides; 4) phragmoplast mediates trafficking of components required for the cell-plate synthesis during cytokinesis. The organization of these arrays requires initiation of new MTs, their elongation, shrinking, severing and bundling with other MTs. Initiation of new MTs occurs on structures called MT-organizing centres (MTOCs) [11]. In animals, centrosomes serve as MTOC during both interphase and cell division. Higher plants lack a conspicuous MTOC and new MTs are nucleated from multiple dispersed sites [12]. A key component of MTOCs is γ-tubulin, an evolutionary conserved homologue protein of α- and β-tubulin [13]–[16]. γ-Tubulin localizes to the MT nucleation sites of interphase and dividing plant cells. There are two γ-tubulin genes in the genome of *Arabidopsis thaliana* and their transcripts were observed in seedlings, roots, flowers and tissue culture cells [17]. Using heterologous expression in fission yeast Horio and Oakley [18] have shown that *Arabidopsis* γ-tubulin was targeted to MTOCs and was able to nucleate MTs. Downregulation or knockout of both genes causes disorganization of cortical microtubule network, spindle and phragmoplast [19], [20]. Thus, plant γ-tubulin plays an essential role in MT organization at all stages of the plant cell cycle.

In active MTOCs, γ-tubulin associates with five proteins forming the γ-tubulin ring complex, or γTuRC [11]. Collectively, six proteins are called γ-tubulin complex proteins (GCPs), with γ-tubulin itself being GCP1. The γTuRC binds to MT minus ends and prevents it from depolymerisation [21]. The *Arabidopsis thaliana* genome contains orthologues of all components of mammalian γTuRC: two γ-tubulin genes (*TUBG1* and *TUBG2*) and γ-tubulin complex protein genes *GCP2* to *GCP6* [22]. GCP2-GCP6 proteins may function as a scaffold for the interaction between 13 γ-tubulin molecules and the MT minus end. Electron microscopy revealed an open ring structure containing γ-tubulin clusters and similar clusters have been found on the minus ends of MTs [23]. In fungal and animal cells, components of the γTuRC preferentially appear at the spindle pole body and the centrosome [24]. In plant cells, γ-tubulin is spatially not restricted to MT ends but also colocalizes along MTs [25], [26] where it nucleates new MTs [27]. Plant GCP proteins are also required for nucleation of MTs. For example two core γTuRC components GCP2 and GCP3 decorate the nuclear envelope of tobacco (*Nicotiana tabacum*) BY-2 suspension cells and are required for MT nucleation and cell division [28], [22], [29]. Recently it has been shown that GCP4 is associated *in vivo* with γ-tubulin in *Arabidopsis thaliana* being an essential component for the function of γ-tubulin in MT nucleation and organization in plant cells [30]. However, the role of other GCP proteins in functional plant γTuRC remains unknown.

The precise coordination of the feeding site establishment and microtubule reorganization suggests that RKN can control the host's cytoskeleton. An example of this control is synchronous assembly of multiple disorganised and enlarged spindles and misaligned phragmoplasts [7]. These phragmoplasts fail to assemble a cell plate and consequently result in the formation of multinucleate cells. Whether these abnormal arrays are simply the remnants of a prematurely aborted cytokinesis or the result of specific rearrangement of MTs and microfilaments in response to parasite factors requires further investigation. However, all data demonstrate that abnormal cytokinesis is principal event for the establishment of the feeding site and successful completion of the nematode life cycle [8].

In order to investigate the role of γ-tubulin in the rearrangements of the cytoskeleton in nematode feeding cells [7] we have carried out nematode infection tests on roots of γ-tubulin mutant lines and show here that knockouts of either gene delays feeding site development. Immunocytochemical analysis show that γ-tubulin protein localizes to the cell cortex and cytoplasm of feeding cells, the nuclear surface and along malformed phragmoplasts. Moreover, γ-tubulin co-localizes and makes a complex with a component of γTuRC, GCP3 [22]. Our data demonstrate that accelerated growth of giant cells and establishment of functional nematode feeding site requires functional γTuRC.

## Results

### Expression of γTuRC Genes in Galls

**Figure 1:**
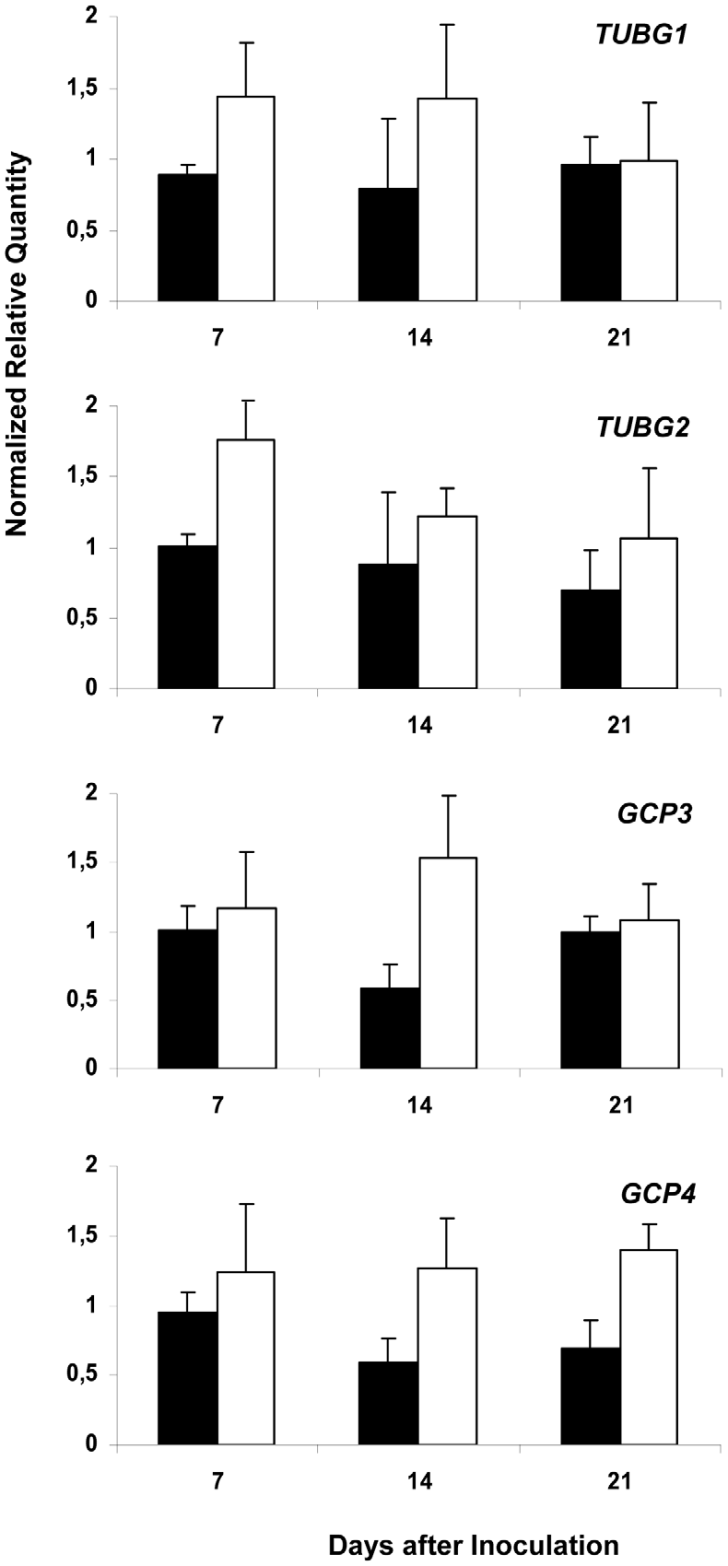
Analysis of Expression Levels of *TUBG1*, *TUBG2*, *GCP3* and *GCP4* in Galls. Relative amount of transcripts of *TUBG1*, *TUBG2*, *GCP3* and *GCP4* genes in *Arabidopsis* galls 7, 14 and 21 DAI (white bars) with *Meloidogyne incognita* by quantitative RT-PCR in comparison to uninfected condition (black bars). All values were normalized according to qBase with the two reference genes At5g62050 and At5g10790, and expressed as normalized relative transcript quantities. The bars are means ±SD of three independent biological replicates.

To explore the role of γTuRC for gall development, we determined transcription levels of four key members (*TUBG1, TUBG2, GCP3* and *GCP4*) by quantitative reverse transcriptase-mediated real-time PCR (qRT-PCR). The total RNA for the assays was extracted from *Arabidopsis* roots infected with *M. incognita* at three stages of gall development: at young stage (7 days after inoculation-DAI), intermediate stage (14 DAI), at a mature stage of gall development (21 DAI) and uninfected roots. Transcription levels augmented in galls for both γ-tubulin genes (*TUBG1* and *TUBG2*) preferentially at early developmental stage (7 DAI) and for two GCPs (*GCP3* and *GCP4*) at intermediate stages (14 DAI) of gall development (Figure 1).

### Nematode Feeding Site Development

**Figure 2:**
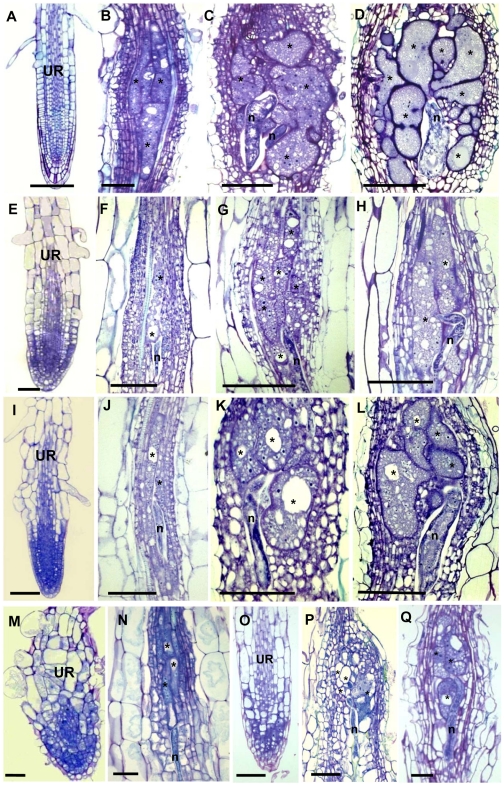
Histological Analysis of Galls and Roots in γ-Tubulin Mutant and Wild-Type *Arabidopsis* seedlings. Bright-field images of sections stained with toluidine blue. **(A)** Uninfected root of wild-type seedlings 40 DAS. **(B)** Gall in wild type roots 7 DAI. **(C)** Gall in wild-type roots 14 DAI. **(D)** Gall in wild-type roots 21 DAI. **(E)** Uninfected root of the γ-tubulin mutant *tubg1-1* seedlings 40 DAS. **(F)** Gall in *tubg1-1* mutant 7 DAI. **(G)** Gall in *tubg1-1* mutant 14 DAI. **(H)** Gall in *tubg1-1* mutant 21 DAI. **(I)** Uninfected root of the γ-tubulin mutant *tubg2-1* seedlings 40 DAS. **(J)** Gall in *tubg2-1* mutant 7 DAI. **(K)** Gall in *tubg2-1* mutant 14 DAI **(L)** Gall in *tubg2-1* mutant 21 DAI. **(M)** Uninfected root of γ-tubulin double mutant *tubg1-1 tubg2-2.* **(N)** Gall in *tubg1-1 tubg2-2* 3 DAI. **(O)** Gall in *tubg1-1 tubg2-2* 7 DAI. **(P)** Gall in amiR-GCP4-9 7 DAI. **(Q)** Gall in amiR-GCP4-9 14 DAI. UR, uninfected root; Asterisks, giant cell; G, gall; n, nematode. Bars = 100 µm **(A)** to **(D)**; 50 µm **(E)** to **(L)**; 20 µm **(M)** to **(Q)**.

**Figure 3:**
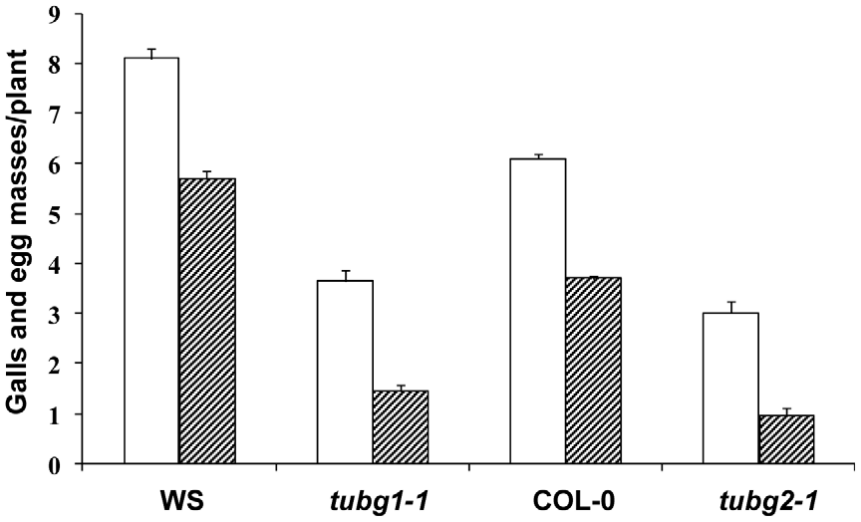
Nematode Infection Test of γ-Tubulin Mutants *tubg1-1* and *tubg2-1* Compared to Wild-Type WS or Col-0. The number of galls (white bars) and egg-masses (lined bars) are significantly decreased in nematode infected roots of both mutant lines compared to wild-type. Data shown represent means±SD from at least two experiments in which a minimum of 60 seedlings of each line were evaluated for nematode infection. Statistically significant differences were determined by the one-way-ANOVA using the SPSS for Windows statistical data analysis package (*P*≤0.05).

To address the functional significance of the upregulation of γTuRC genes during feeding site development, we investigated the morphology of galls at different developmental stages in 4 mutant lines (*tubg1-1*, *tubg2-1*, *tubg1-1 tubg2-2* and amiR-*GCP4-9*) (Figure 2). Infection tests were performed to evaluate the competence of nematode development and reproduction in mutant lines (Figure 3). Cortical, epidermal and root hair cells of uninfected roots, of *tubg1-1* and *tubg2-1* of mature seedlings (40 days after sowing-DAS) were swollen, expanding isotropically (Figure 2A, 2E and 2I) but no perceptible phenotype was seen in the vascular tissue where galls develop. This phenotype was not detected in young roots (14 DAS) used for nematode infection (Figure S1; wild-type root 1A and mutant lines 1B to 1D) and nematode penetration or infection occurred normally (Figure S1E). Lateral root development also appeared similar in both wild-type and mutant lines. A lack of root phenotype in roots of both γ-tubulin mutant lines was also observed by Pastuglia *et al*. [20]. Based on these observations we predict that the mild phenotype present in mature uninfected roots (40 DAS) should not influence on gall development. The abnormalities of root morphology and cells expansion were more pronounced in the double mutant (Figure 2M). Although, knockdown of γ-tubulin genes had no discernable effect on the ability of nematodes to penetrate, migrate, and induce giant cells, feeding sites development was delayed at 7 DAI in both mutant lines (Figures 2B, 2F and 2J). At 14 DAI giant cells in γ-tubulin mutants were smaller and contained enlarged vacuoles (Figures 2C, 2G and 2K). At this stage nematodes often remained vermiform at stage 2 juvenile (J2) whereas in wild-type parasitic J2 were larger. At 21 DAI, wild-type plants contained typical multinucleated cells with dense cytoplasm (Figure 2D). In contrast, the infected roots of γ-tubulin mutant lines had smaller feeding cells (Figures 2H and 2L) with less nuclei and large vacuoles. The development of a fraction of nematodes was arrested at the parasitic juvenile stages. In the double mutant line (*tubg1-1 tubg2-2*) infection process resulted in tiny giant cells containing an average of two nuclei (Figures 2N). Uninfected roots of the GCP4 mutant line did not show any evident phenotype (Figure 2O) except for shorter root hairs. Young giant cells in the amiR-*GCP4-9* line were smaller than in wild-type, contained a reduced number of nuclei and enlarged vacuoles (Figure 2P). At later stages of development (14 DAI and 21DAI) nematode development was delayed and giant cells remained small (Figure 2Q at 14 DAI). Examination of giant cell morphology correlates with nematode reproduction. Assessment of the nematode life cycle in γ-tubulin mutant lines (*tubg1-1* and *tubg2-1*) showed arrest of half of galls development and delayed nematode maturation. Consequently, production of egg masses was significantly reduced (Figure 3). A similar analysis could not be performed with the double mutant since the plants are viable for only 3 weeks.

### Localization of γ-Tubulin in Nematode Feeding Cells

**Figure 4:**
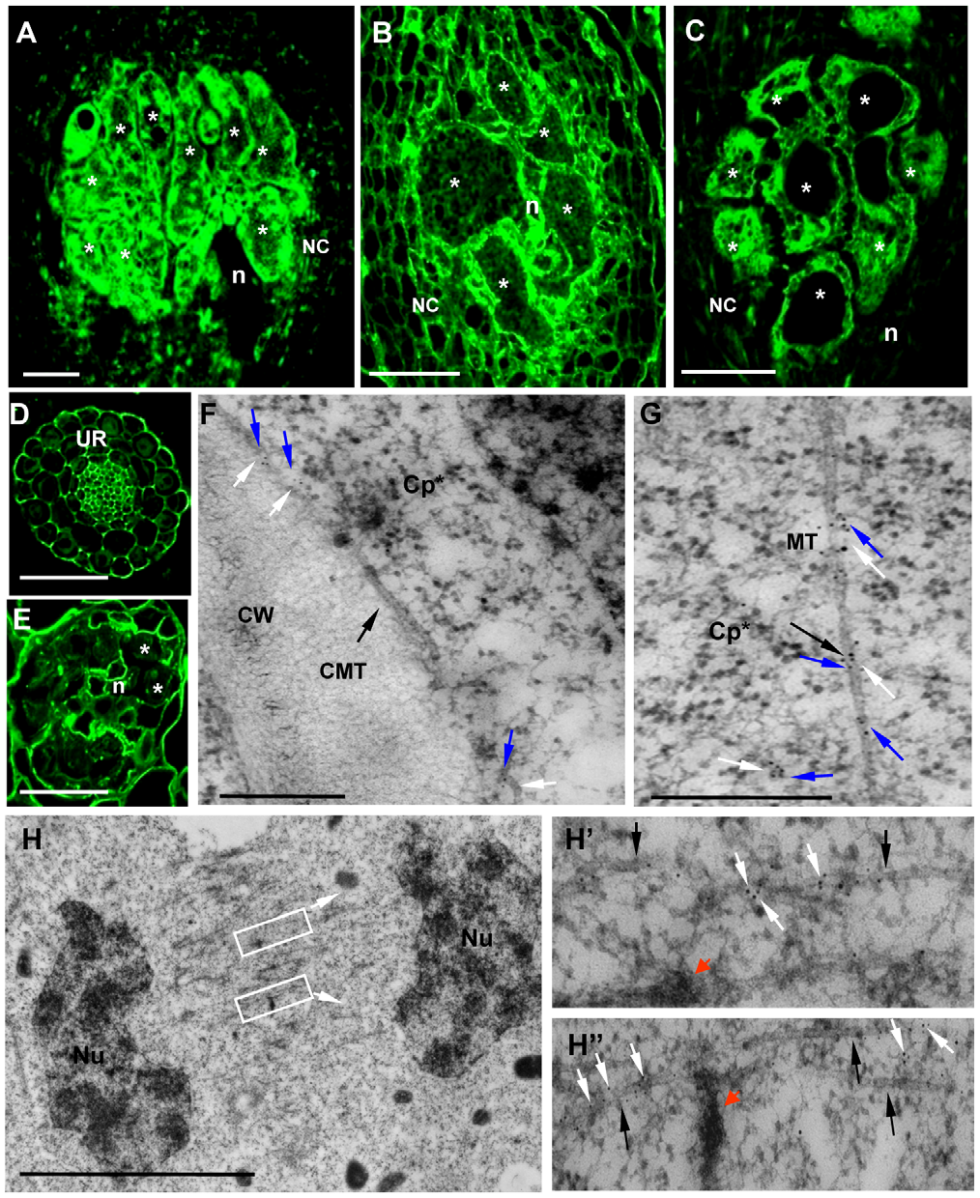
Immunofluorescence Detection of γ-Tubulin on Galls and Roots in Mutants and Wild-Type *Arabidopsis* seedlings. Galls 14 DAI of wild-type (A), of *tubg1-1* (B), of *tubg2-1* (C). Uninfected root of wild-type seedling (D). Gall 7 DAI of *tubg1-1 tubg2-2* (E). Dissected galls (14 DAI) were sectioned and processed for double immunoelectron microscopy with anti-γ- and α-tubulin primary antibodies, followed by secondary 10 and 5 nm gold-conjugated antibody respectively. (F) γ-tubulin (white arrows) is localized with cortical MT (black arrow) and associated with α-tubulin (blue arrows). (G) γ-tubulin binds to cytoplasmic MT. (H), (H') and (H'') γ-tubulin is dispersed throughout the misaligned phragmoplast MTs and co-localizes with α-tubulins. Fragments of cell wall are visible at MT ends as dark patches (red arrows) possibly inducing failure in giant cell cytokinesis. Asterisks, giant cell; n, nematode, UR, uninfected root; NC, neighboring cells; CMT, cortical microtubule; Cp* giant cell cytoplasm; CW, cell wall; MT, microtubule; Nu, nucleus. Bars = 50 µm in (A) to (E), 500 nm in (F) and (G) and 5 µm in (H).

The localization of γ-tubulin in galls of wild-type and mutant lines was analysed by immunocytochemistry (Figure 4) using a polyclonal antiserum [20]. In order to locate both γ-tubulin proteins separately in galls, we used the mutant *tubg1-1* for TUBG2 localization and , *tubg2-1* for TUBG1 detection. In wild-type and mutant roots (*tubg1-1*, *tubg2-1* and *tubg1-1 tubg2-2*) γ-tubulin staining was observed in all root cells (Figure 4D). At 14 DAI galls of wild-type seedlings, γ-tubulin protein was localized throughout the giant-feeding cells and fewer label was detected in the neighbouring cells (Figure 4A and Figure S2A and S2D). At the same stage of infection, roots of *tubg1-1* revealed γ-tubulin staining in the giant-cell cortex and less in the cytoplasm (Figure 4B and Figure S2B and S2E) while in *tubg2-1* mutant γ-tubulin was localized along the giant cell cortex and around the nuclei (Figure 4C and Figure S2C and S2F). Weak γ-tubulin expression was seen in giant cells of *tubg1-1 tubg2-2* (Figure 4E).

Immuno-gold analysis of the infected wild-type plants (14 DAI) by electron microscopy demonstrate that γ-tubulin co-localize with MTs (α-tubulin) in the giant cell cortex (Figure 4F), cytoplasm (Figure 4G), at the nuclear surface (Figure S3B and S3B') and with the phragmoplast during mitosis (Figure 4H to 4H'). Cell wall fragments were visible at the MT ends as dark patches of severely misaligned phragmoplasts (Figure 4H and 4H'). Free cytosolic γ-tubulin is apparent in Figure S3A.

### Spindles in Root Cells of γ-Tubulin Mutants of *A. thaliana* Are Curved

Crosses between γ-tubulin mutant lines (*tubg1-1* and *tubg2-1)* and marker lines expressing *Pro35S*-*MBD:GFP* and nuclear histone *H2B:YFP* were used to study microtubule organization in the mutant background (Figure S4). Mitotic spindles were bowed and chromosomes were often misaligned in both γ-tubulin mutant lines (Figure S4A for *tubg1-1* and 4B, 4C for *tubg2-1*) in comparison with wild-type root cells (Figure 4D).

### GCP3 Co-localizes with γ-Tubulins in Nematode Feeding Cells

**Figure 5:**
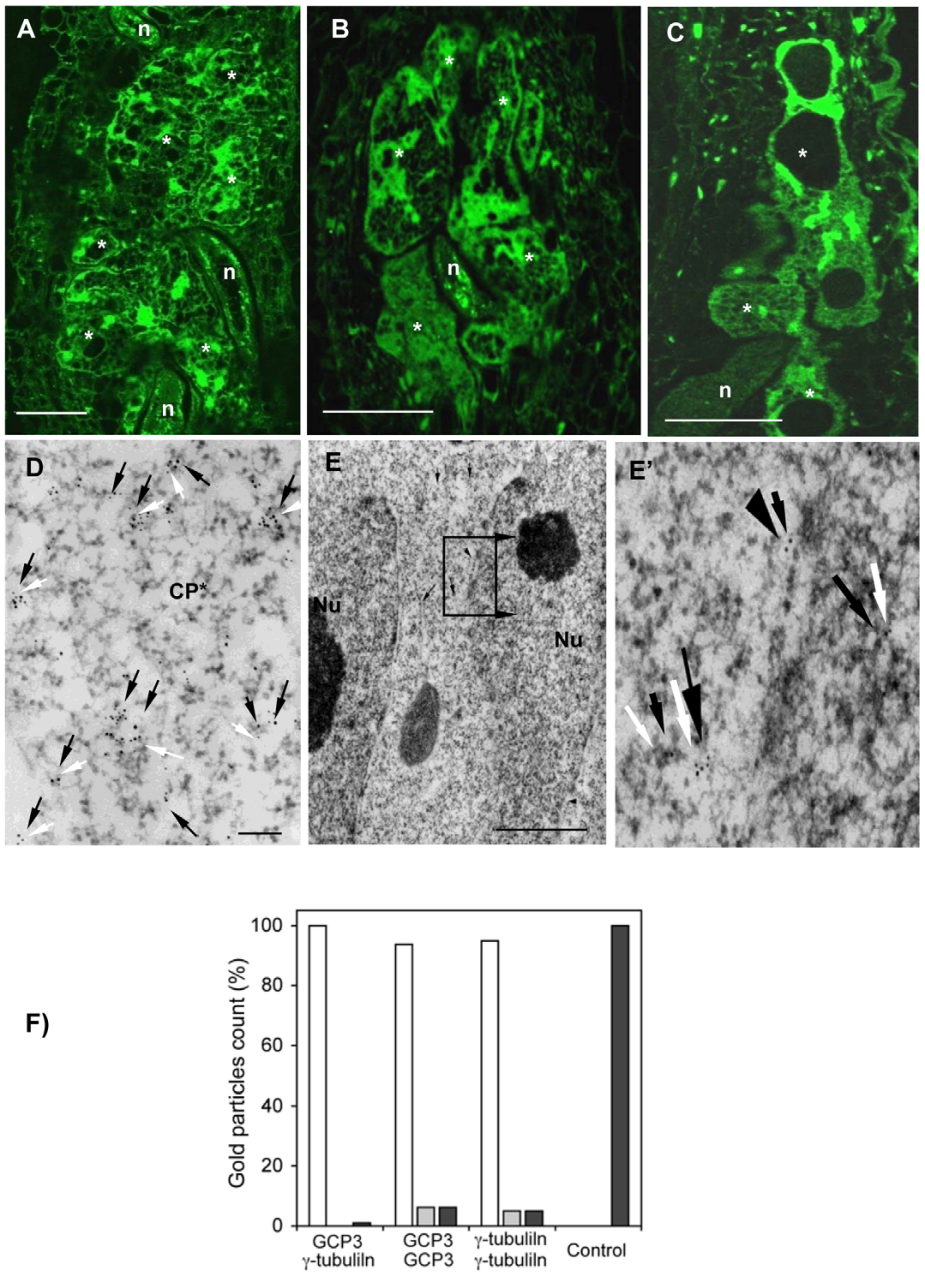
γ-Tubulin Complex Protein 3 (GCP3) is Present in Giant Cells and Co-localizes with γ-Tubulin. **(A)** to **(C)** Immunostaining of GCP3 (green) in galls 14DAI of wild-type and mutant lines. Gall in wild-type roots **(A)**. Galls in *tubg1-1* roots **(B)**. Galls in *tubg2-1* roots **(C)**. Dissected wild type galls (14 DAI) were sectioned and processed for double immunoelectron microscopy with anti-GCP3 and γ-tubulin primary antibodies, followed by secondary 10 and 5 nm gold-conjugated antibody respectively. GCP3 (black arrows) and γ-tubulin (white arrows) co-localize in the cytoplasm **(D)** and at the nuclear surface **(E, E')**. Both proteins are also present as monomers in the cytoplasm. **(F)** Histogram illustrating distances between gold particles showing that GCP3 and γ-tubulin are often in proximity less than 10 nm suggesting their interaction. White bars are for distances less then 10 nm, grey for distances between 10 and 50 nm, and black bars for distances above 50 nm. Asterisks, giant cell; n, nematode; Nu, nucleus; Cp*, giant cell cytoplasm. Bars = 50 µm **(A)** to **(C)**; 100 nm **(D)**; 300 nm **(E)**.

GCP3 localization was observed in giant cells of wild-type (Figure 5A) as well as *tubg1-1* (Figure 5B) and *tubg2-1* (Figure 5C) plants concentrating around the nuclei and cell cortex. Co-localization of GCP3 and γ-tubulin was analysed using electron microscopy. Both proteins were found in the cytoplasm (Figure 5D), at the nuclear surface (Figure 5E and 5E'), and at the cell cortex (Figure S5A and S5A'). Commonly, multiple colloidal gold particles corresponding to γ-tubulin and GCP3 grouped together forming compact clusters in the cytoplasm (Figure 5D). We have measured the distance between closest gold particles and found that majority of them were in the proximity of less than 10 nm (Figure 5F). These data provide compelling evidence for the interaction between GCP3 and γ-tubulin in giant cells as part of a single multi-protein complex.

### Analysis of *TUBG1-GFP* Localization in Uninfected and Nematode Infected Roots

**Figure 6:**
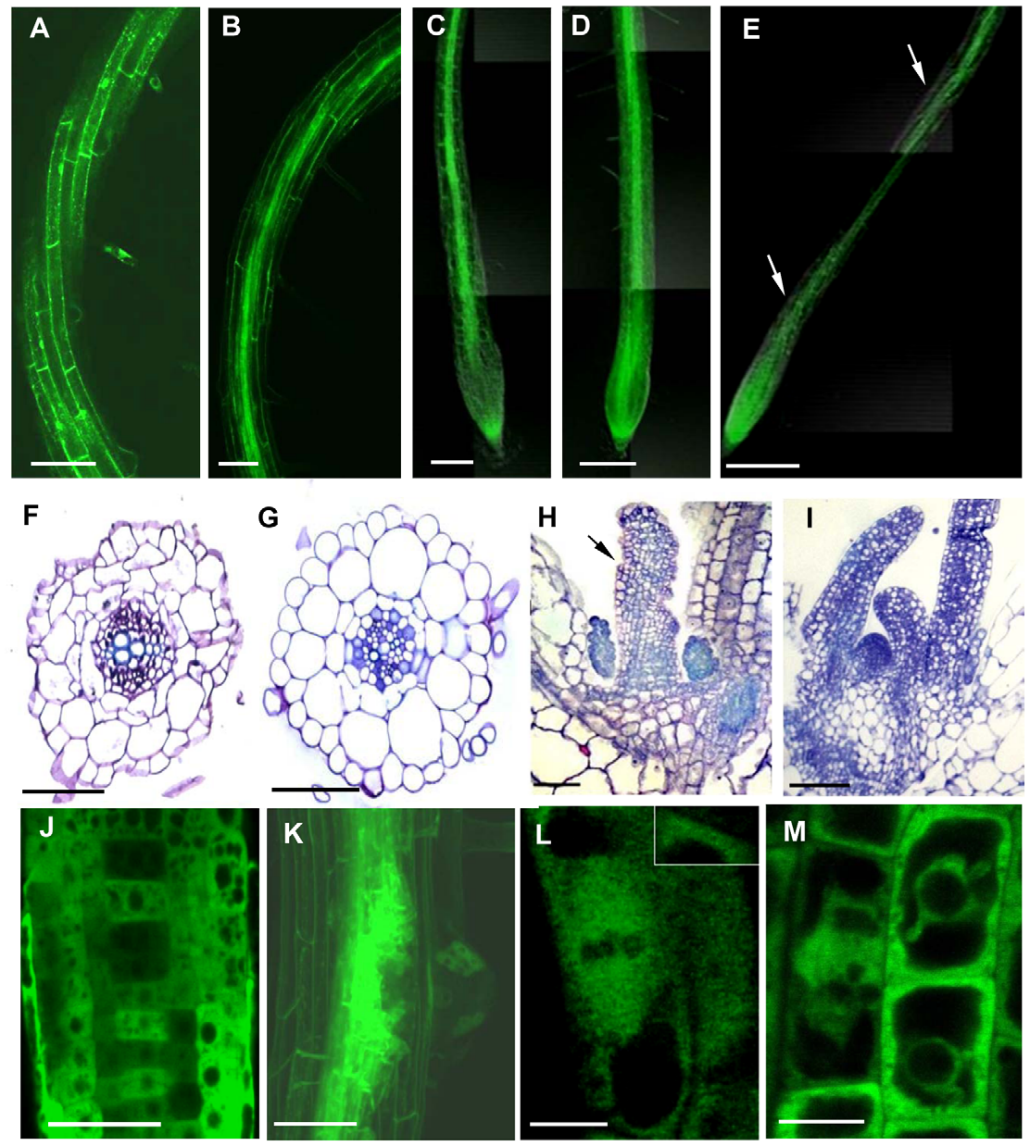
Ectopic Expression of γ-Tubulin in *Arabidopsis thaliana* Seedlings Causes Root Twisting and Leaf Curling. γ-Tubulin overexpressing roots show twisted phenotype **(A)** and **(B)**. Roots treated with either oryzalin (**C)** or propyzamide **(D)** did not show root twisting as observed (arrows) in the γ-tubulin overexpressing untreated roots **(E)**. Cross section of a γ-tubulin overexpressing root stained with toluidine blue showed miss-shaping of the root cells and disorganization of the root tissues **(F)** compared to the wild-type **(G)**. Longitudinal section of a shoot apical meristem stained with toluidine blue showing a leaf curling phenotype (black arrow) of γ-tubulin overexpressing seedlings **(H)** compared to the wild-type **(I)**. γ-tubulin localization (green) in the root elongation zone **(J)** and lateral root meristem showing a patchy expression pattern **(K)**. γ-tubulin localization along a spindle **(L)** and a phragmoplast **(M)**. Bars = 100 µm in **(A)** to **(E)**; 50 µm **(F)** to **(I),** **(J)** and **(K);** 20 µm **(L)** and **(M)**.

*35Spro:TUBG1-GFP* and *35Spro:GFP-TUBG1* were transiently expressed in tobacco leaf cells using *Agrobacterium* infiltration. GFP fluorescence was observed in the *Arabidopsis* cytoplasm and in the nucleus for both constructs (Figure S6) and discrete fluorescent dots were apparent in the cytoplasm and cell cortex. *Arabidopsis* seedlings were transformed with *35Spro:TUBG1-GFP* and *35Spro:GFP-TUBG1* constructs and the F1 generation was analyzed for GFP expression. Seedlings containing the C-terminal fusion (*TUBG1-GFP*) showed the best germination efficiency. The roots of seedlings germinated on the vertical plates exhibit skewing to the left if observed from the top side of the plate (Figure S6A) and the leaves were curling (Figure S7C) compared to wild-type (Figure S7B and S7D respectively). Confocal microscopy imaging of roots revealed a left-handed twisting phenotype (Figure 6A and 6B). The twisting is likely to result from the miss-shaping of the root cells and disorganization of the root tissues as shown in a cross section (Figure 6F compared to 6G). Sections of a shoot apex (Figure 6H compared to 6I) showed right-handed displacement of the cells. To verify if twisting was caused by the altered properties of MTs we treated γ-tubulin overexpressing seedlings with MT polymerisation inhibitors propyzamide and oryzalin. Both treatments inhibited root skewing and left-handed twist of the epidermal cell layers implying that ectopic expression of γ-tubulin increase stability of the microtubules leading to the organ twisting phenotype (Figure 6C, 6D compared to a twisted root in 6E). Plants expressing γ-tubulin-GFP lacking a twisted phenotype but still showing GFP-fluorescence were used for localization studies. Transgenic root apical meristem and lateral root meristem displayed a patchy expression pattern (Figure 6J and 6K) that varied between different roots suggesting a post-transcriptional control of γ-tubulin expression. In mitotic cells γ-tubulin was localized to the spindle (Figure 6L) and the phragmoplast (Figure 6M). During the interphase, multiple discrete foci and homogeneous fluorescence were observed in the cytoplasm (Figure 6L inset and 6M).

We investigated the mobility of γ-tubulin in the cytoplasm using fluorescence loss in photobleaching (FLIP) analysis. Most of the γ-tubulin-GFP fusion protein was found to be highly mobile (Figure S8) suggesting that only a minor fraction of total γ-tubulin is utilised in the nucleation of microtubules.

**Figure 7:**
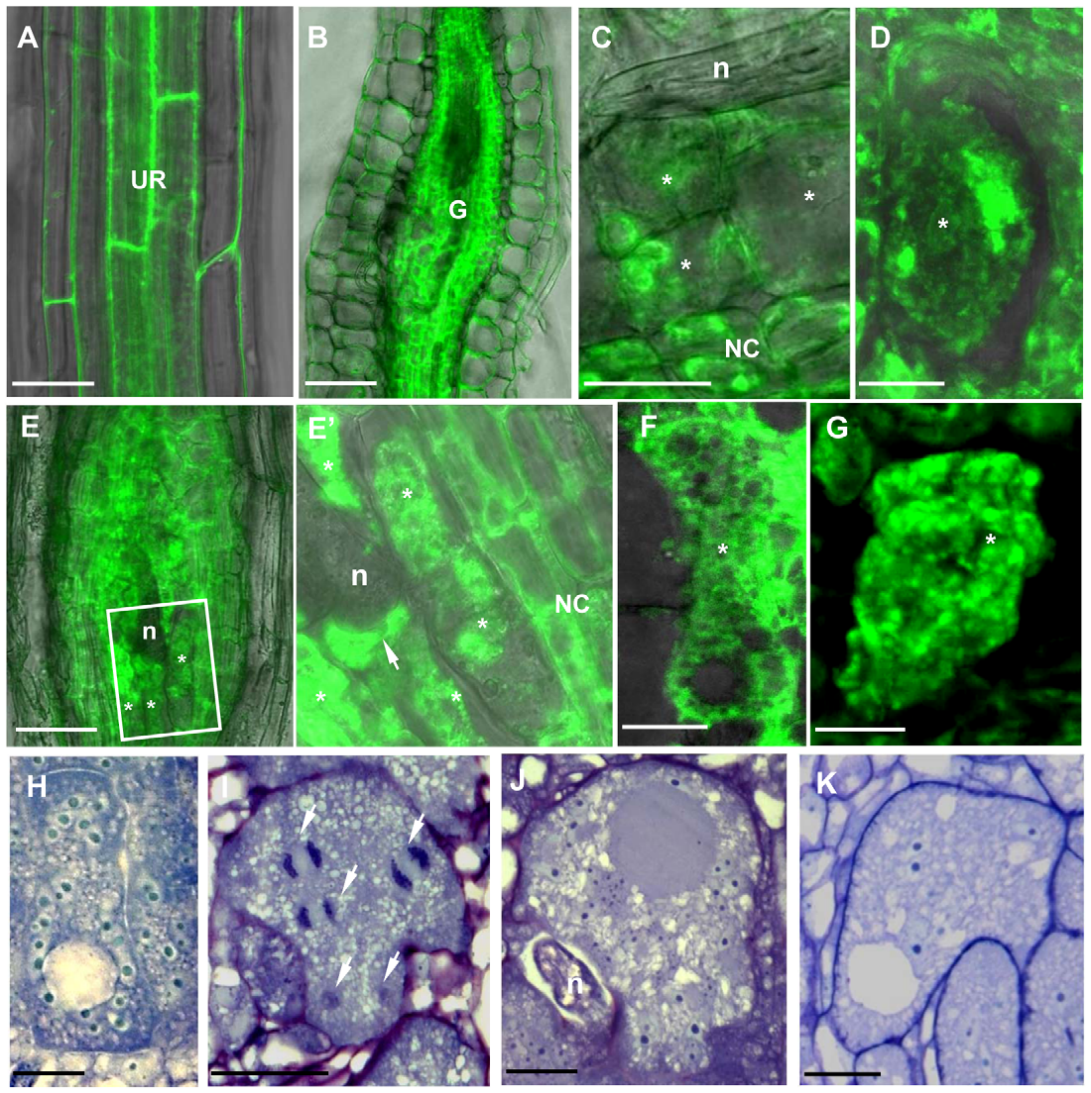
γ-Tubulin Localization and Overexpression in Giant Cells of Nematode Infected Roots of the *TUBG1-GFP* line. **(A)** γ-Tubulin localization in an uninfected root. **(B)** γ-Tubulin localization in a whole gall. **(C)** γ-Tubulin localization in young giant cells 3 DAI **(D)** and 5 DAI. **(E)** γ-Tubulin localization in a whole gall 7 DAI and **(E')** detail of a giant cell showing accumulation of γ-tubulin protein close to the nematode head (arrow). **(F)** γ-Tubulin localization in a giant cell 10 DAI. **(G)** γ-Tubulin localization around the nuclei of a giant cell 7 DAI. **(H)** Giant cell overexpressing γ-tubulin 7 DAI. **(I)** Mitotic events in a giant cell (white arrows) overexpressing γ-tubulin 14 DAI. **(J)** Giant cell overexpressing γ-tubulin 21 DAI. **(K)** Giant cell in a wild-type gall 14 DAI. UR, uninfected root; Asterisks, giant cell; G, gall; NC, neighbouring cells; n, nematode, Nu, nucleus. Bars = 50 µm in **(A)** and **(B)**, 20 µm in **(C)**, **(E)**, **(H)** and **(K)**, 10 µm in **(D)**, **(G)**, **(I)** and **(J)**, 5 µm **(F)**.

γ-Tubulin-GFP fluorescence was also observed in the cells of uninfected root vasculature (Figure 7A) as well as throughout all gall tissues (Figure 7B). Young giant cells (3 DAI and 5 DAI) showed concentration of γ-tubulin around the nuclei and diffuse fluorescence in the cytoplasm (Figure 7C and 7D). At the later stages (7 DAI and 10 DAI) γ-tubulin expression was observed in all gall and giant cells (Figure 7E, 7E' and 7F) and a speckled fluorescence was notable around the perinuclear cytoplasm (Figure 7G).

**Figure 8:**
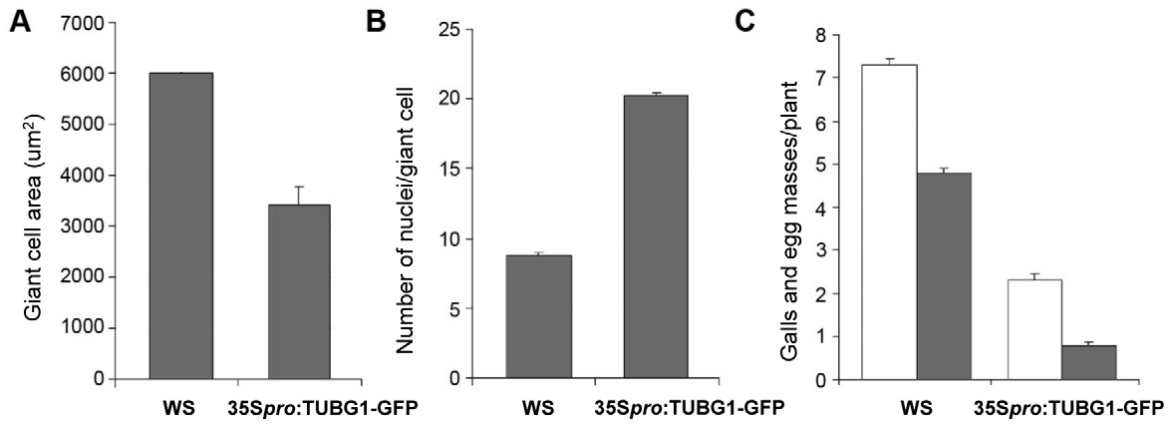
Giant Cell Area, Number of Nuclei and Infection Tests of γ-Tubulin Mutants Compared to Wild-Type. Giant cell area **(A)** and number of nuclei **(B)** in roots under ectopic expression of γ-tubulin compared to wild-type and nematode infection test **(C)** of roots under ectopic expression of γ-tubulin. Area was measured on 60 giant cells. Number of nuclei was counted on 60 giant cells. The number of galls (white bars) and egg-masses (lined bars) are significantly decreased under ectopic expression of γ-tubulin compared to wild-type. Data shown represent means±SD from at least two experiments in which a minimum of 60 seedlings of each line were evaluated for nematode infection. Statistically significant differences were determined by the one-way-ANOVA using the SPSS for Windows statistical data analysis package (*P*≤0.05).

Young (7 DAI; Figure 7H) as well as transitional and mature giant cells (14 DAI and 21 DAI; Figure 7I and 7J) remained small and contained more nuclei than wild-type control (Figure 7K). Mitotic chromosomes were detectable in giant cells of γ-tubulin overexpressing lines (Figure 7I), but not in the wild-type (Figure 7K). Area measurements on giant cells confirmed a decreased size compared to wild-type (Figure 8A). Nuclei counts per section at the core of giant cells (7 DAI) validated the observation of a larger number of nuclei per giant cell when under ectopic γ-tubulin expression (Figure 8B). Infection tests have shown a decrease in gall number and egg mass production in γ-tubulin overexpressing lines (Figure 8C).

## Discussion

### Role of γ-Tubulin and GCP3 in Nematode Infection

Transcription analyses demonstrate augmentation of the γ-tubulin (*TUBG1* and *TUBG2*) and two γ-tubulin-complex protein genes (*GCP3* and *GCP4*) in the course of infection with root-knot nematodes in galls. This corroborates with *in situ* hybridization analysis which has shown the transcriptional activation of the γ-tubulin genes in giant feeding cells as well as in neighbouring tissues that derived from *M. incognita* targeted vascular root cells [7]. The transcription levels of *TUBG1* and *TUBG2* were higher at an early stage of gall development (7 DAI) while *GCP3* and *GCP4* transcription increased at an intermediate stage (14 DAI). The transcription of three genes (*TUBG1, TUBG2* and *GCP3*) declined in mature giant cells (21 DAI) coinciding with the end of the mitotic activity in giant-feeding cells. In addition to γ-tubulin proteins, both GCP3 and GCP4 are indispensable components for the MT nucleating activity of γTuRC in plant cells [22], [30]. This suggests that an optimum level of γ-tubulin and GCP in galls is necessary to provide a sufficient number of new microtubule nucleation sites for the remodelling of the MT network in giant-feeding cells and to support recurrent ongoing mitotic activity in both giant- and neighbouring cells [7].

Analysis of the T-DNA knockout lines demonstrates the importance of γ-tubulin for the establishment of the feeding site and completion of the parasite life cycle. No visible phenotype was observed in the vasculature of single knockout plants before infection by the root-knot nematodes in agreement with the partial functional redundancy of *TUBG1* and *TUBG2* proposed by Pastuglia *et al*. [20] and Binarova *et al*. [19]. Both double mutant *tubg1-1*-*tubg2-2* and RNAi knockdown plants exhibit disruption of anisotropic cell expansion [19], [20] in a similar manner to the drug-induced reduction of the number of nucleation sites and randomization of cortical MTs [31]. Although nematodes were able to penetrate, migrate, and induce gall formation in roots of *tubg1-1* and *tubg2-2* lines, there was a significant delay in feeding site formation, indicating that both γ-tubulin genes are required for proper nematode feeding site development. Consequently, the number of nematodes that could complete their life cycle and reproduce was significantly lower in both T-DNA lines as compared to wild-type. It has been demonstrated in γ-tubulin knockout lines that reduced MT nucleation can delay chromosome separation and nuclear proliferation, thereby inhibiting cell division and impairing growth polarity [32]. We have observed that residual levels of γ-tubulin in the double knockout line is sufficient for gall induction in agreement with the dose-dependent effect of γ-tubulin on the early feeding site development and underlines the functional significance of a rise of γ-tubulin gene transcription.

Localization of γ-tubulin throughout giant cell differentiation illustrates its role during nematode infection. At the early stages of gall development (3 DAI) γ-tubulin is mainly localized at the nuclear surface in giant-feeding cells as well as neighbouring cells. As giant cells matured, a scattered GFP fluorescence was also detected in the cytoplasm and cell cortex. Accumulation of γ-tubulin close to the nematode head suggests reorganization of the microtubule network in the region proximal to the secretion and/or feeding processes, consistent with the accumulation of ER and other organelles at this site (de Almeida Engler, unpublished data). At this stage, nematodes are alternately injecting secretions and feeding on the cell cytoplasm. It is known that the cytoskeleton acts differently depending on the plant host and the invading pathogen, and that the MT response to infection is variable between different plant-microbe interactions [33], [34]. So far, the molecular mechanisms regulating MT dynamics during host/parasite interactions is not well understood and our data suggest that increased γ-tubulin expression might be part of this apparatus.

### γ-Tubulins Co-localize with MT and GCP3

Immunolocalization experiments demonstrate that γ-tubulin and GCP3 co-localize each other and MTs around the nuclei, in the cell cortex and in mitotic MT arrays. Therefore dispersed free or MT associated γTuRCs in giant cells might provide nucleation of new MTs required for fast array reorganization in giant cells. γ-Tubulin was shown to associate with preprophase bands (PPBs), spindle, phragmoplast and cortical cytoplasm of soybean, onion, *Arabidopsis* and cells of other species [17], [25], [35], [36], [37]. Each MT array appears to have multiple sites for the nucleation of new MTs which can be located along the lattice of extant MTs, resulting in branching of cortical MTs. This activity may result in the array composed of randomly oriented bundles observed in the giant cell cortex. In addition, γ-tubulin cooperates with other known regulators of MT organization including MOR1/GEM1 and MAP65 to regulate MT organization in giant cells [8], [38], [39], [40], [41].

Although γ-TuRC components are conserved in plant genomes, their association in a functional complex has not yet been proven. The pioneering study of Seltzer *et al*. has provided biochemical evidence for the association of *Arabidopsis* GCP2, GCP3 and γ-tubulin in the cytoplasmic soluble complex [22]. Recently Nakamura *et al*. [42] have shown that all six members of the *Arabidopsis* γ-TuRC immunoprecipitate jointly. Immunogold electron microscopy shows that γ-tubulin and GCP3 are located less then 10 nm apart from each other proving further evidence for the existence of γ-TuRC *in vivo* and its close association with the MT lattice.

Both GCP2 and GCP3 localize at the nuclear envelope and play a role in MT nucleation [22], [28] and GCP2 can control organization of cortical MTs by positioning the γ-tubulin-containing complex on pre-existing MTs [29], [43]. The presence of abnormal spindles in uninfected *Arabidopsis* roots of *tubg1-1* and *tubg2-2* lines agrees with the previous observations of collapsed or defective spindles and chromosome segregation defects in γ-tubulin mutants of several species such as *S. pombe*, *S. cerevisiae* and *Drosophila melanogaster* [14], [44], [45], [46], [47]. In addition, γ-tubulin depletion in *A. nidulans* abolishes nucleation of spindle MTs [48]. This suggests that γ-tubulins have conserved functions in organizing the spindle in phylogenetically distant organisms. Furthermore, a reduction of the γ-tubulin signal seen on spindle and phragmoplast in amiR-GCP4 cells suggests their interaction with these arrays during mitosis in plant cells [30].

### Overexpression of γ-Tubulin Induces Stability of Microtubule Network

The localization of γ-tubulin-GFP in living root cells corroborates with results obtained by immunostaining. Indeed, a strong GFP signal was observed on mitotic spindles and phragmoplasts during mitosis as well as during interphase around nuclei, a known site of MT nucleation. The apparent speckled fluorescence in the cytoplasm indicates the presence of discrete γTuRCs. A similar distribution pattern has been observed in BY-2 cells transiently expressing a GFP-γ-tubulin fusion protein. There, at the end of cell division γ-tubulin was firstly accumulated at the daughter nuclear surfaces and evenly spread along the cell cortex [49], [50]. In animals, γ-tubulin is also important for the coordination of late mitotic events [51] and has a MT-independent function in mitotic checkpoint control [52]. Whether plant γ-tubulin performs similar functions remains unknown, but it can not be ruled out that reduction of nuclei number in the giant cells of the double mutant results from the deficiency in these activities.

Our FRAP analysis indicate that the absolute majority of γ-tubulin-GFP freely distributes in the cytoplasm supporting the idea that microtubule nucleation events in plants are driven by γ-tubulin-containing complexes released from the nucleation sites and redistributed around the cell. Electron microscopy observations confirm the cytoplasmic distribution of γ-tubulin and the presence of a large number of closely associated γ-tubulin and GCP3 proteins pointing towards their interaction.

Analysis of the ectopic γ-tubulin expression in plants shows a curling phenotype in leaves and skewing of roots, suggesting an alteration in dynamics and/or organization of MT arrays. Treatment of seedlings with oryzalin and propyzamide restored the normal pattern indicating that overexpression of γ-tubulin leads to MT stabilization or excessive nucleation. It is also plausible that ectopic expression of γ-tubulin in giant cells provokes higher mitotic activity by accelerating nuclear division. In addition, excessive nucleation of MTs might impede giant cell expansion, nematode feeding and interfere with gall development and eggmass production. Our previous reports suggested that cytoskeleton stabilization by drug treatment (taxol) or ADF2 downregulation can disturb gall development and consequently nematode maturation [7], [9].

### Microtubule Dynamics in Giant Cells and Concluding Remarks

**Figure 9:**
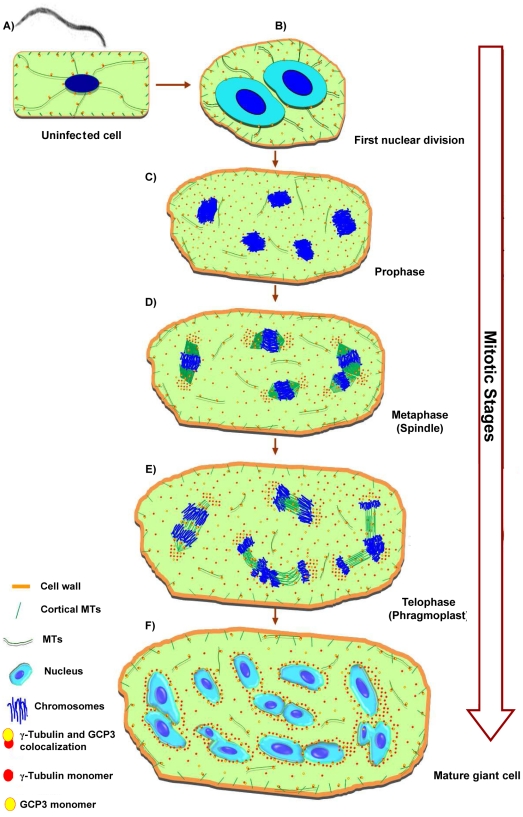
Microtubule Organization in Giant Cells. The model of giant cell cytoskeleton reorganisation is based on observations of a large number of gall sections. The illustrations do not precisely reflect the total number of chromosomes or nuclei effectively present per giant-cell. Root-knot nematodes invade root cells and induce vascular cells **(A)** to become giant-feeding cells **(B)** to **(F)**. The first visible symptom of nematode infection on the microtubule cytoskeleton of a young giant cell is the increase in density of tubulins in the cytoplasm. At this stage, the first nuclear division results into two enlarged nuclei with outsized nucleoli **(B)**. Giant cells contain a dense cytoplasm and scarce cytoplasmic microtubules which co-localize with GCP3 protein. Young giant cells contain a dense network of randomly distributed cortical microtubules bound to γ-tubulins, and GCP3 **(A)** to **(F)**. During giant cell expansion the cytoplasm contain scarce microtubules, and γ-tubulins and GCP3 proteins. The cytoplasmic microtubules remain disarrayed throughout giant cell development. Mitotic giant cells harbour nuclei containing a large number of condensed chromosomes often dividing in synchrony **(C)** to **(E)**. During prophase nuclei are often clearly separated containing their packed chromosomes **(C)**. In the course of metaphase to telophase spindles are large and malformed **(D).** Accumulation of γ-tubulin and GCP3 occurs mainly around the chromosomes and on the spindles. γ-tubulin and GCP3 localize to giant cell phragmoplasts which are misaligned and fail to expand centrifugally resulting in aborted cytokinesis **(E)**. Some nuclei appear to show incomplete division or to have fused **(F)**. The density of cortical microtubules is reduced and and cytoplasmic microtubules are sparse. Mature giant cells finally present multiple lobed nuclei which recurrently appear connected to each other, are surrounded by γ-tubulin and GCP3 proteins which are often co-localized suggesting the presence of MTOCS at these sites of giant cells. Our data suggests that γ-tubulin and GCP3 recruitment contributes to microtubule nucleation in mitotic and cortical arrays in root-knot nematode feeding cells.

Taking into account present and previous data [7] we propose a model for MT dynamics in giant cells (Figure 9). The transcription analysis shows that two components of plant MTOCs (γ-tubulin and GCPs) are highly expressed in galls. Knockout of either *TUBG1* or *TUBG2* genes results in inhibition of gall and nematode development, demonstrating that both proteins are important for feeding site formation, while in non-infected plants gene redundancy has been observed [20]. Predominant localization of TUBG2 at the giant cell cortex compared to a more cytoplasmic distribution of TUBG1 suggests that they might exert different functions within these large feeding cells. Double knockout completely abolishes gall development. The reduction of γ-tubulin protein level compromises the MT network integrity. Our previous findings demonstrate the significance of MT and actin filaments during interphase as well as mitosis [7]-[9] for successful nematode infection and reproduction. γ-tubulin is crucial for the organization and function of mitotic spindle and phragmoplast.

The overall effect of one or both γ*-tubulin* and the *GCP4* genes knockout on the MT network of giant cells agrees with the hypothesis that γTuRC proteins including γ-tubulins and GCP4 are required for proper functioning of mitotic arrays and MT nucleation. Bowed spindles observed in root tip cells can be caused by abnormal nucleation of MTs. Since unusually shaped and enlarged spindles are normally observed in giant cells, these anomalies might be caused by the unbalance between γ-tubulin and GCP3 concentration in giant cells, ultimately resulting in aneuploid nuclei that failed to divide [5], [53]. High γ-tubulin and GCP3 concentration may play a role in the formation of disarrayed phragmoplasts observed in giant-feeding cells and might contribute to the misalignment of the cell plate and arrest in giant cell division.

The apparent fragmentation and reduction in density of interphase MTs in giant cells could result from defective nucleation. Nucleation, dynamics, and spatial organization of MTs are tightly coordinated processes in plant cells. Proteins secreted by nematodes may induce MT reorganization by altering MT dynamics. Up-regulation of γTuRC proteins in galls provides an excess of MT nucleation sites, which in combination with other factors might control MT dynamics leading to the reorganization of the entire array by site-specific destabilization/stabilization activity.

Microtubule response to pathogen invasion depends on the type of plant–microbe interactions [33] and nematodes are the only known pathogens capable of inducing the long-term cytoskeleton rearrangements on their host plants. The local reduction of density of the cytoplasmic MTs may facilitate susceptibility of host plants to nematodes in a similar manner to other systems. For example, microtubule depolymerisation at the infection site has been observed in the soybean and parsley cells attacked by the oomycete *Phytophthora sojae* [54], [55]. Stabilization of MTs by taxol blocks gall development, while breakdown permits nematode reproduction [7]. Although what stimulates cytoskeletal responses in nematode feeding cells is still not known, MT and actin rearrangements might be directly or indirectly induced by effectors secreted by the nematodes still to be identified.

Here we show that a tight balance between MT nucleation and dynamics is required for the successful nematode infection and γTuRC is essential to exercise this balance. The knockout of the individual components of γTuRCs reduces the efficiency of MT nucleation and consequently affects gall development and inhibits nematode reproduction, while the overexpression of γ-tubulin causes overall stabilisation of the MT network and produces a similar effect on the nematode life cycle. Since upregulation of MT nucleation in developing giant-feeding cells is essential for nematode parasitism, the components of γTuRC can be envisaged as potential targets to design alternative strategies to control pathogen invasion and spread.

## Materials and Methods

### Plant Material, Growth Conditions, Nematode Inoculation and Infection Tests

T-DNA mutagenized lines of the two γ-tubulin genes of *Arabidopsis thaliana* were obtained from the Versailles and SALK collections as described by Pastuglia *et al*. [20]. The CVP11 (*tubg1*-*1*) and T628 (*tubg2-2*) lines have been acquired from a T-DNA–mutagenised population of the ecotype Wassilevskija (WS) from Pastuglia *et al*. [56]. The SALK_004612 (*tubg2-1*) line was acquired from the ABRC and originates from a T-DNA–mutagenised population of the Columbia-0 (Col-0) ecotype [57]. Surface-sterilized seeds of WS, Col-0 and γ-tubulin knockdown lines were germinated on Murashige and Skoog medium (Duchefa, Haarlem, the Netherlands) containing 1% sucrose and 0.8% plant cell culture–tested agar (Sigma-Aldrich) and 50 mg/mL kanamycin for T-DNA mutagenised lines. After 10 days under growth chamber conditions of 16-h/8-h light/dark cycles at 25°C kanamycin resistant seedlings were transferred to Knop medium which favors root development [58]. Plates were inclined at an angle of 60° to allow the roots to grow along the surface to facilitate harvesting of roots and galls. For nematodes inoculation, around 100 surface-sterilized, freshly hatched second stage juveniles (J2s) of *Meloidogyne incognita* were adjusted on each 14 day old seedling as previously described [9]. For nematode infection tests seedlings were kept in Knop medium during 60 days to allow nematodes to complete their life cycle. At 14 days after inoculation galls were counted and after 60 days egg mass numbers were scored.

### Quantitative RT-PCR

Total RNA of non-meristematic roots and galls of *A. thaliana* cv. WS dissected at various time points after nematode inoculation (7, 14, 21 DAI) was extracted according to the procedure described by Laroche-Raynal *et al*. [59]. One microgram of high-quality RNA was reverse-transcribed using the iScript cDNA Synthesis Kit (Bio-Rad, Marnes-la-Coquette, France). Amplification and detection were performed in the Opticon 2 system (MJ research, Bio-Rad). Reaction mixtures were of a final volume of 15 µL, containing 7.5 µL qPCR MasterMix Plus For SYBR Green I No Rox (Eurogentec, Angers, France), 0.13 µM of each primer and 5 µL of 50-fold diluted cDNA templates. PCR conditions were as follows: 95°C for 15 min, followed by 40 cycles of 95°C for 15 s, 58°C for 30 s and 72°C for 30s. At the end of the programme, a melting curve (from 65 to 95°C, read every 0.5°C) was determined to ensure that only single products were generated. At5g10790 and At5g62050 were used for normalization of qRT-PCR data. These two genes were previously identified as showing constant expression in response to different biotic stimuli [60], [61], [62]. Raw data were treated using the MJ Opticon Monitor Analysis software (version 3.1; Bio-Rad). Quantifications were performed with the modified ÄCt method employed by the qBase1.3.5 software, and expressed as normalized relative quantity. The primer list is given in Table S1. Three independent quantitative RT-PCR reactions were carried out per sample and three biological replicates were performed. The bars represent mean values from three independent experiments.

### Morphological Analysis

For morphology observation of uninfected roots and galls of γ-tubulin knockout lines compared to control WS and Col-0 (*tubg1-1* line was made in WS and *tubg2-1* in Col-0; 20), samples grown *in vitro* were fixed in 2% glutaraldehyde in 50 mM PIPES buffer, pH 6.9, and then dehydrated and embedded in Technovit 7100 (Heraeus Kulzer) as described by the manufacturer. Embedded roots and gall tissues were sectioned (3 µm) and stained in 0.05% toluidine blue and mounted in Depex (Sigma-Aldrich). Microscopic observations were performed using bright-field optics and images were performed with a digital camera (Axiocam, Zeiss).

### Immunofluorescence Analyses of γ-Tubulin, α-Tubulin and GCP3

Uninfected roots and feeding sites of roots, inoculated with J2s of *M. incognita*, of *A. thaliana* cv. WS, Col-0, *tubg1-1*, *tubg2-1*, and *tubg1-1 tubg2-2* mutant lines were fixed in 4% formaldehyde in 50mM Pipes buffer (pH 6.9). Dissected galls (7, 14, and 21 DAI) were dehydrated and embedded in butyl-methylmethacrylate as described by Kronenberger *et al*. [63] with some modifications. The immunolocalization procedure was performed essentially as described by de Almeida-Engler *et al*. [7]. Slides containing sectioned nematode feeding sites were incubated with acetone absolute for 30 min to remove the plastic. Primary and secondary antibodies were diluted 1∶300 and 1∶500 (v/v) respectively, in blocking solution (1% bovine serum albumin in 50 mM Pipes, pH 6.9). Sections were incubated with blocking solution for 30 min, and overnight at 4°C with the primary antibodies. As controls, sections were incubated with pre-immune serum or without primary antibodies. Anti-γ-tubulin and anti-GCP3 were generated respectively as described by Pastuglia *et al*. [20] and Seltzer *et al*. [22]. First, incubation with a polyclonal anti-γ-tubulin or anti-GCP3 has been performed and slides were then washed in Pipes buffer for 15 min. Slides were subsequently incubated for 2 h at 37°C with the secondary antibody anti-rabbit Alexa 488 (green fluorescence) or Alexa 594 (red fluorescence) and washed in Pipes buffer for 15 min. For DNA visualization sections were stained with 4′, 6-diamidino-2-phenylindole (DAPI) (Sigma-Aldrich) at 1 µg/ml in Pipes buffer. Slides were mounted in 90% glycerol in ddH2O and were observed with a microscope (Axioplan 2; Zeiss) equipped for epifluorescence microscopy and differential interference contrast optics, and images were collected with a digital Axiocam (Zeiss).

### Immunoelectron Microscopy of Galls and Juvenile Nematodes

Root galls of *A. thaliana* cv. WS and Col-0 were dissected at 14 DAI after inoculation and fixed in a mixture of 1.5% glutaraldehyde, 3% paraformaldehyde in 10 mM PBS containing 150 mM NaCl (pH 7.2) for 3 h at room temperature. After several washes in PBS buffer fixed galls were incubated in 0.5 M NH_4_Cl for 1 h, dehydrated in graded ethanol series, embedded in acrylic resin LR White (Sigma), and polymerized overnight at 60°C. Ultrathin sections were collected on parlodion-coated nickel grids, treated with 0.1 M HCl for 10 min, and washed at least twice with bidistilled water. The grids were pre-incubated in 1% bovin serum albumin (BSA) in PBS for 15 min, prior to incubation with pre-immune goat serum (Sigma) diluted 1∶10 in BSA-PBS for 1 h. Immuno-labelling was performed with primary antibodies diluted with BSA-PBS. Double labelling was done by treatment of grids either with rabbit polyclonal anti-γ-tubulin (1∶500) and the monoclonal anti-α-tubulin (1∶500), for 90 min at room temperature. The grids were washed 3 times for 5 min in BSA-PBS and incubated for 1 h with secondary antibodies (10 nm gold-goat anti-rabbit and 5 nm gold-goat anti-mouse antisera; British BioCell International) diluted 1∶20 with BSA-PBS. Other grids containing gall sections were incubated in the following immunoreagents: first, grids were incubated with rabbit polyclonal antiserum anti-GCP3 diluted in BSA-PBS. Samples were washed 3 times for 5 min in BSA-PBS and incubated for 1 h with the secondary antibody (10 nm gold-goat anti-rabbit). Secondly, grids were incubated with the rabbit polyclonal antibody against γ-tubulin (1∶300), washed 3 times for 5 min in BSA-PBS and incubated for 1 h with the secondary antibody goat anti-rabbit gold (5 nm). All grids were rinsed 3 times for 5 min in PBS, rinsed in bidistilled water and stained 5 min in 2% aqueous uranyl acetate and 2 min in 1% lead citrate. Samples were observed under a Philips 400 T electron microscope.

### *In vivo* Observations on Whole Roots and Fresh Nematode Infected Root Slices and Confocal Microscopy

Observation of MTs and nuclei in the nematode feeding sites in wild-type and mutant lines was performed in nematode infected *A. thaliana* seedlings harbouring the MT binding domain of *MAP4* fused to *-GFP* and H2B *Histone-YFP* (*MBD::GFP- His::YFP*). More than 50 root meristems and young galls (2 to 7 DAI) were directly observed under the microscope. At least 50 galls at various time points after infection (7 to 15 DAI) were dissected from roots and embedded in 5% agar. Fresh thick sections of 50–100 µm (7 to 10 DAI) or 150–200 µm (10 to 14 DAI) were performed with a HM650V vibrotome Microm (Walldorf, Germany). Whole roots and fresh slices were observed using an inverted confocal microscope (model LSM510 META; Zeiss). YFP and GFP fluorescence were monitored in Lambda mode with a 499 to 550 nm beam path (488 nm excitation line). The fluorescent dye Syto-82 (Molecular Probes) was used at 1 mM final concentration. GFP or YFP and Syto-82 fluorescence were monitored in Lambda mode using the 488 nm argon laser line excitation and spectral detection using 10 nm steps for emission between 499 to 620 nm. All observations were obtained from at least three independent experiments.

### Constructing TUBG1::GFP and Plant Transformation

The complete coding sequences of *TUBG1* gene was amplified by PCR, using ecotype cDNAs as the template, the primers pairs attB1F [5′-AA AAA GCA GGC TTC-(ACC ATG)-(18/25 nt template-specific seq)-3′] and attB2R [5′-A GAA AGC TGG GTG (TTA)-(template-specific seq)-3′] with adapter primers attB1F (5′-GGGACAAGTTTGTACAAA AAAGCAGGCT-3′) and attB2 R (5′-GGGGACCACTTTGTACAAGAAAGCTGGGT-3′). These sequences were then inserted into plant expression vectors, using Gateway™ Technology (Invitrogen) and the pDONR207 donor vector (Invitrogen). Cloning was carried out in *Escherichia coli* DH10β cells. Transient expression of γ-tubulin in tobacco leaves was performed in leaves of *Nicotiana benthamiana* by infiltrating *Agrobacterium tumefasciens* strain C58C1 harbouring the *35Spro:TUBG1-GFP* and *35Spro:GFP-TUBG1* with a syringe and observations were performed 48 hours after infiltration on the confocal microscope (Zeiss, LSM 510). To generate transgenic plants expressing the γ-tubulin protein the *35Spro:TUBG1-GFP* (*TUBG1-GFP*) plasmid was transformed into *Agrobacterium tumefasciens* strain C58C1 and *Arabidospsis thaliana* Col-0 were transformed by floral dipping [64]. Plants with γ-tubulin-GFP fluorescence which did not present a visible phenotype and showing less expression were used for localization studies. On the other hand, seedlings with root twisting harbouring γ-tubulin-GFP fluorescence and showing additional γ-tubulin-GFP expression were used for overexpression studies.

### Oryzalin and Propyzamide Treatments

To test the effect of MT cytoskeleton inhibitors on the γ-tubulin overexpressing line presenting twisted roots, *TUBG1-GFP* seeds were germinated on MS medium, transferred to the same medium supplemented with oryzalin (0.5 µM) or propyzamide (5 µM) and incubated overnight at room temperature. Control experiments were performed using the same medium in the absence of inhibitors. Treated and untreated roots were imaged with a confocal laser scanning microscope (LSM510 META; Zeiss) using the tile-scan mode with the 10x/0.3NA objective. GFP fluorescence was recorded using 488-nm laser excitation and the 505–530 nm BP emission filter.

### Accession Numbers

*Arabidopsis* Genome Initiative locus identifiers for the genes mentioned in this article are as follows: At3g61650 (*TUBG1*), At5g05620 (*TUBG2*), At5g06680 (*GCP3*) and At3g53760 (*GCP4*).

## Supporting Information

Figure S1 Histological Analysis of Roots in γ-Tubulin Mutants and Wild-Type *Arabidopsis* Seedlings. Bright-field images of sections stained with toluidine blue. Bars = 50 µm. **(A)** Uninfected root of wild-type. **(B)** Uninfected root tip and **(C)** vascular tissue of the γ-tubulin mutant *tubg1-1.* **(D)** Uninfected root of γ-tubulin mutant *tubg2-2.* **(E)** Infected root of γ-tubulin mutant *tubg2-2* showing massive nematode (arrows) penetration and migration in a mutant root.
(TIF)

Figure S2 Immunofluorescence Detection of γ-Tubulin in Galls in Mutants and Wild-Type *Arabidopsis* Seedlings. Galls 14 DAI of wild-type **(A, D)**, of *tubg1-1* **(B, E)**, of *tubg2-1* **(C, F)**. γ-Tubulin fluorescence is in green in **(A)**, **(B)** and **(C)** and in red in **(D)**, **(E)** and **(F)**. Asterisks, giant cell; n, nematode. Bars = 50 µm.
(TIF)

Figure S3 Electron Micrographs Show γ-Tubulin and α-Tubulins Localization in Giant Cells. Dissected galls (14 DAI) were sectioned and processed for double immunoelectron microscopy with anti-γ- and α-tubulin primary antibodies, followed by secondary 10 and 5 nm gold-conjugated antibody respectively. **(A)** Monomeric γ- (white arrows) and α-tubulins (blue arrows) are distributed in the cytoplasm. **(B)** and **(B')** γ- and α-tubulins are apparent at the nuclear surface. n, nematode; NC, neighboring cell; Asterisks, giant cell; CW, cell wall; Cp* giant cell cytoplasm; Nu, nucleus. Bars = 1 µm **(A)** and 10 µm **(B)**.
(TIF)

Figure S4 Mitotic Cell in Roots Cells of γ-Tubulin Mutants of *A. thaliana* Exhibit Curved Spindles and Misaligned Chromosomes. Projections of serial confocal optical sections of mutant lines co-expressing the microtubule binding domain MBD::GFP (green) and nuclear histone H2B::YFP (red) proteins. **(A)** Spindle in root cells of *tubg1-1;* **(B, C)** of *tubg2-1* and **(D)** in wild-type. Bars = 5 µm.
(TIF)

Figure S5 Double Immunoelectron Microscopy Localization of GCP3 and γ-Tubulin in Giant Cells. Dissected wild type galls (14 DAI) were sectioned and processed for double immunoelectron microscopy with anti-GCP3 and γ-tubulin primary antibodies, followed by secondary 10 and 5 nm gold-conjugated antibody respectively. **(A** and **A')** GCP3 co-localizes with γ-tubulin to the cell cortex. n, nematode; Cp*, giant cell cytoplasm; Cu, nematode cuticle; CW, cell wall. Bars = 300 nm **(A)**.
(TIF)

Figure S6 Localization of Transiently Expressed *TUBG1-GFP* in Leaf Pavement Cells *Nicotiana tobaccum*. **(A)** C-terminal fusion, *TUBG1-*GFP; and **(B)** N-terminal fusion *GFP-TUBG1*. Bar = 20 µm.
(TIF)

Figure S7 Ectopic Expression of γ-Tubulin in *Arabidopsis thaliana* Seedlings Causes Skewed Roots and Curled Leaves. Roots of γ-tubulin overexpressing seedlings skew to the left side **(A)** compared to wild type **(B)**; γ-tubulin overexpressing seedlings exhibit leaf curling **(C)** compared with the wild-type **(D)**.
(TIF)

Figure S8 FLIP Analysis of γ-Tubulin-GFP on a Root Apical Meristem Cell of *Arabidopsis thaliana*. **(A)** Beginning of the photobleach: region of interest 1 (ROI 1; red) shows γ-tubulin-GFP in the mitotic spindle; ROI 2 (green) is a cytoplasmic region proximal to the bleach region ROI 4 (yellow). ROI 3 (violet) is a cytoplasmic region distant from the bleach region. ROI 5 (blue) is a neighbouring cell used as control. **(B)** Late stages of the photobleach show bleaching of the majority of GFP-γ-tubulin molecules (see graph to the left). ROI 1 (red) corresponds to the spindle position with remaining fluorescence above the background of ROI 3 (violet) and ROI 2 (green), suggesting a fraction of Sγ-tubulin bound to the spindle has a slow turnover. (C) Image representing 6 time points of the fluorescence intensity distribution in meristematic root cells following a FLIP regime of 16 bleach repeats of each 10secs. Arrowhead points to a spindle.
(TIF)

Table S1 Primer Sequences Used in qRT-PCR Amplification of the *AtTUBG1*, *AtTUBG2, AtGCP3*, *AtGCP4*, *AtOXA1* and *AtUBP22* Gene Transcripts.
(DOCX)
