# Supplementary figures and images for: Feeding Cells Induced by Phytoparasitic Nematodes Require γ-Tubulin Ring Complex for Microtubule Reorganization
Source: PLoS Pathog. 2011 Dec 1;7(12):e1002343. doi: 10.1371/journal.ppat.1002343 (PMC3228788; doi:10.1371/journal.ppat.1002343)

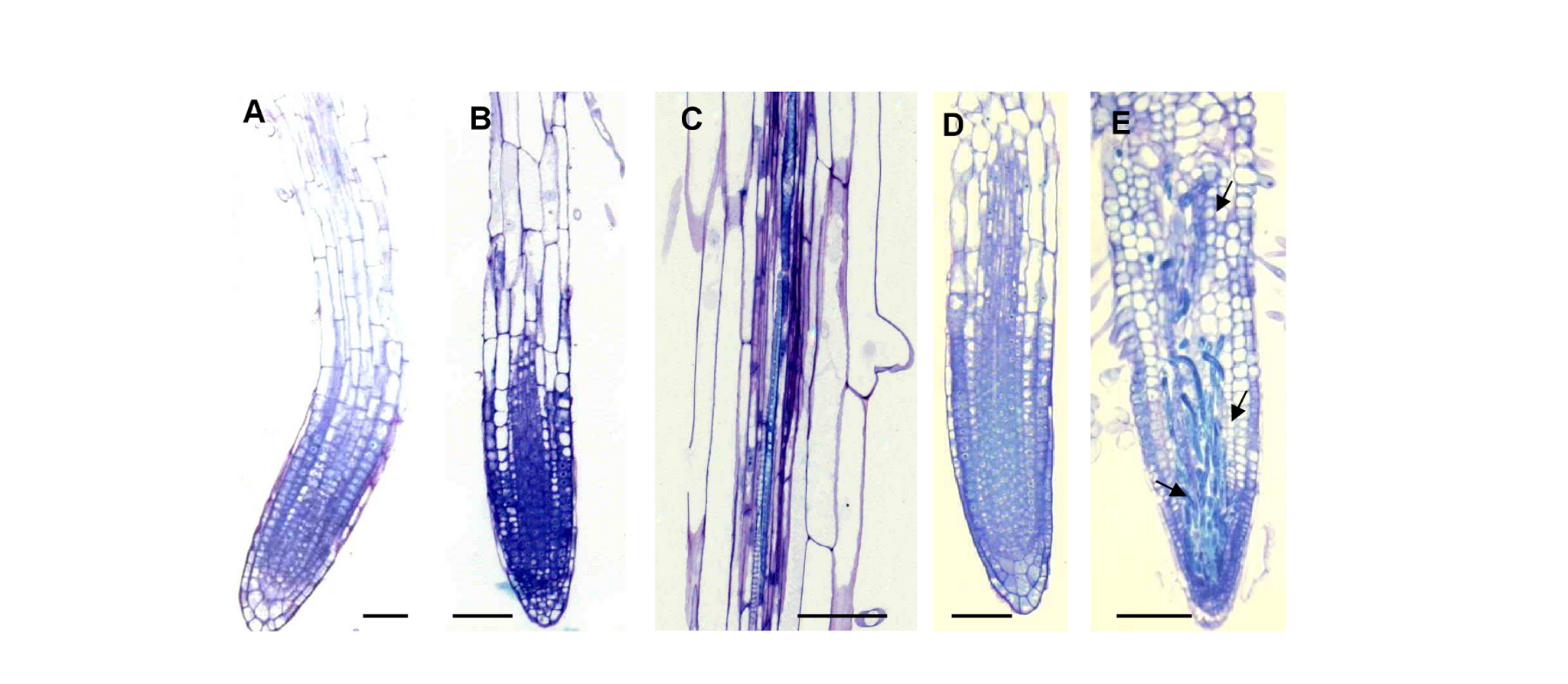

Supplement: Figure S1 — Histological Analysis of Roots in γ-Tubulin Mutants and Wild-Type Arabidopsis Seedlings. Bright-field images of sections stained with toluidine blue. Bars = 50 µm. (A) Uninfected root of wild-type. (B) Uninfected root tip and (C) vascular tissue of the γ-tubulin mutant tubg1-1. (D) Uninfected root of γ-tubulin mutant tubg2-2. (E) Infected root of γ-tubulin mutant tubg2-2 showing massive nematode (arrows) penetration and migration in a mutant root. (TIF) [file ppat.1002343.s001.tif]

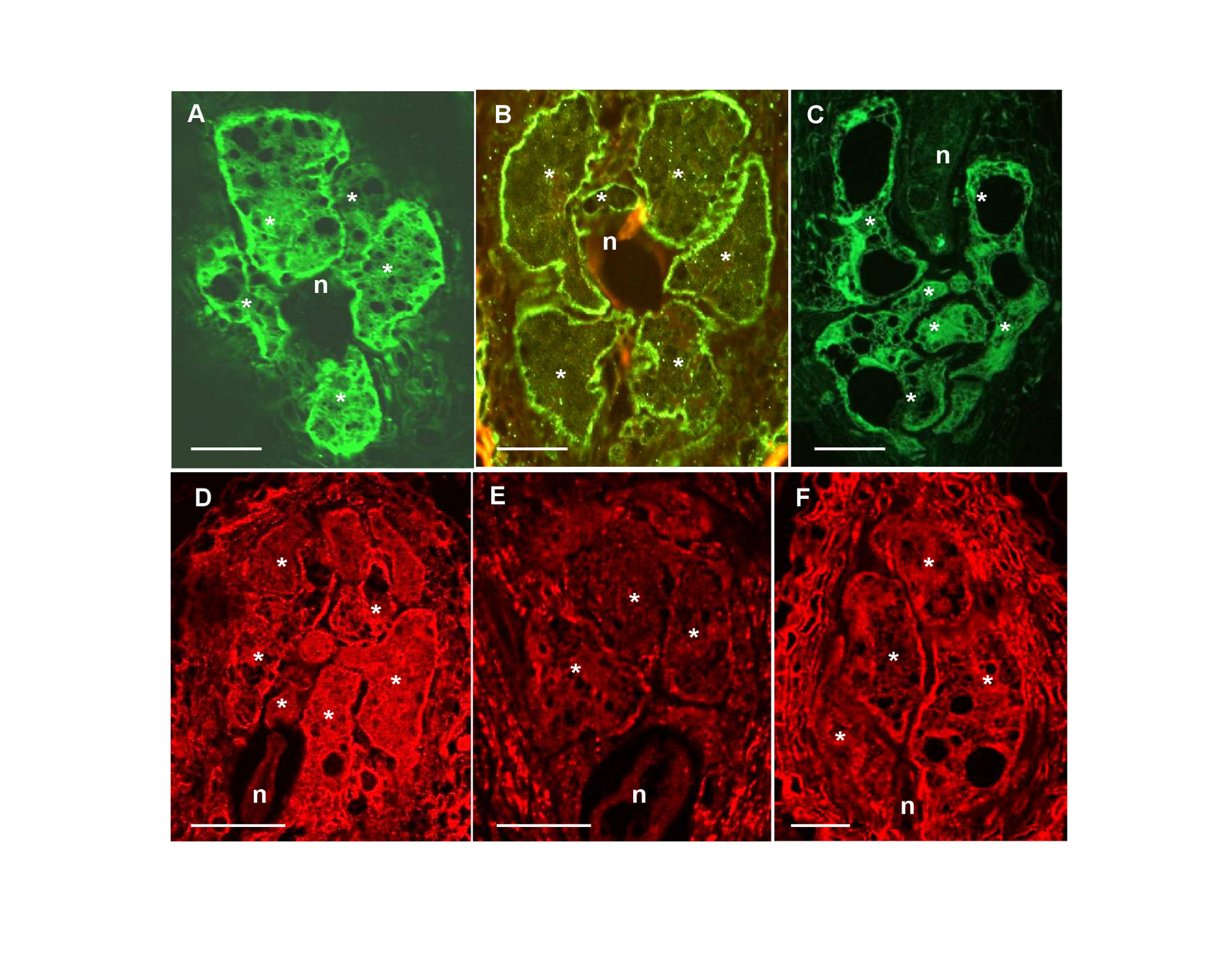

Supplement: Figure S2 — Immunofluorescence Detection of γ-Tubulin in Galls in Mutants and Wild-Type Arabidopsis Seedlings. Galls 14 DAI of wild-type (A, D), of tubg1-1 (B, E), of tubg2-1 (C, F). γ-Tubulin fluorescence is in green in (A), (B) and (C) and in red in (D), (E) and (F). Asterisks, giant cell; n, nematode. Bars = 50 µm. (TIF) [file ppat.1002343.s002.tif]

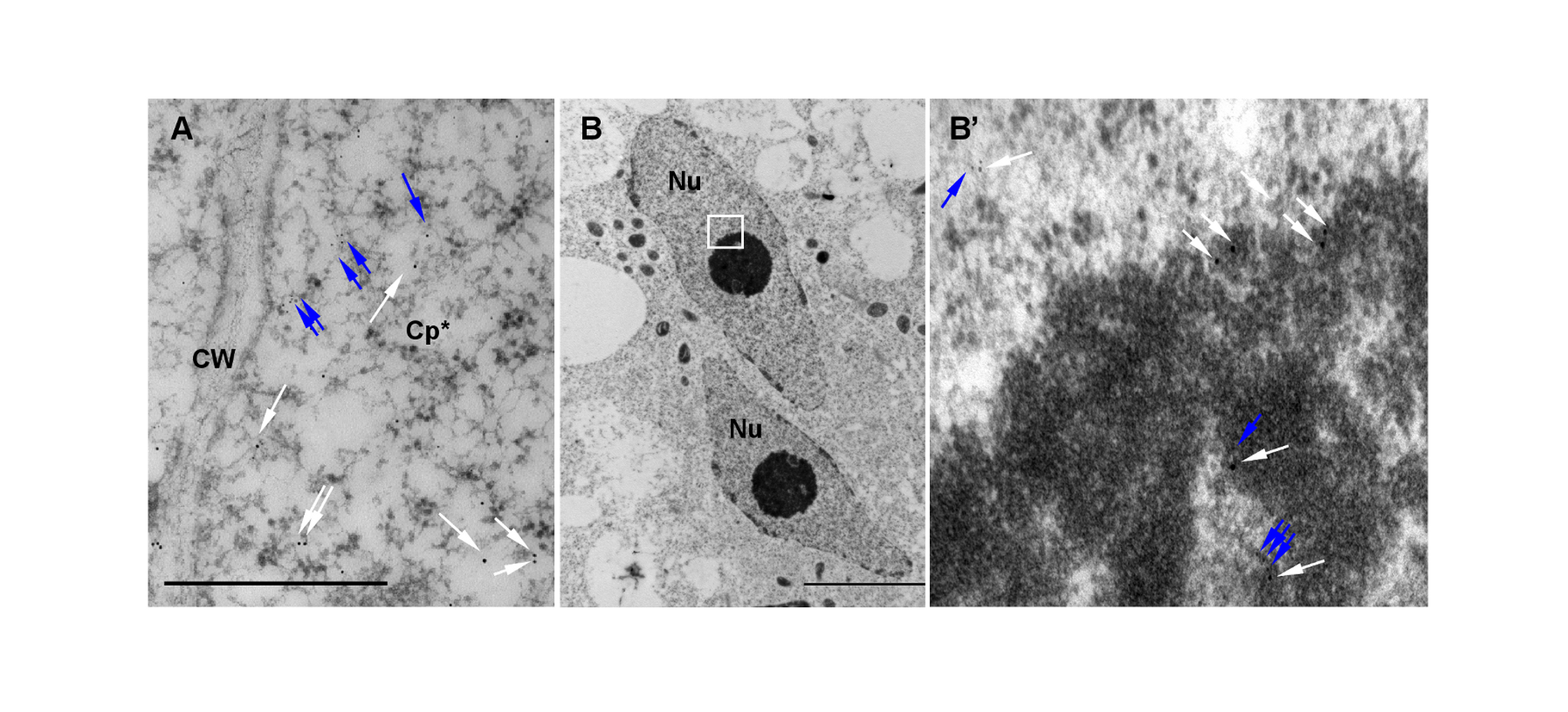

Supplement: Figure S3 — Electron Micrographs Show γ-Tubulin and α-Tubulins Localization in Giant Cells. Dissected galls (14 DAI) were sectioned and processed for double immunoelectron microscopy with anti-γ- and α-tubulin primary antibodies, followed by secondary 10 and 5 nm gold-conjugated antibody respectively. (A) Monomeric γ- (white arrows) and α-tubulins (blue arrows) are distributed in the cytoplasm. (B) and (B') γ- and α-tubulins are apparent at the nuclear surface. n, nematode; NC, neighboring cell; Asterisks, giant cell; CW, cell wall; Cp* giant cell cytoplasm; Nu, nucleus. Bars = 1 µm (A) and 10 µm (B). (TIF) [file ppat.1002343.s003.tif]

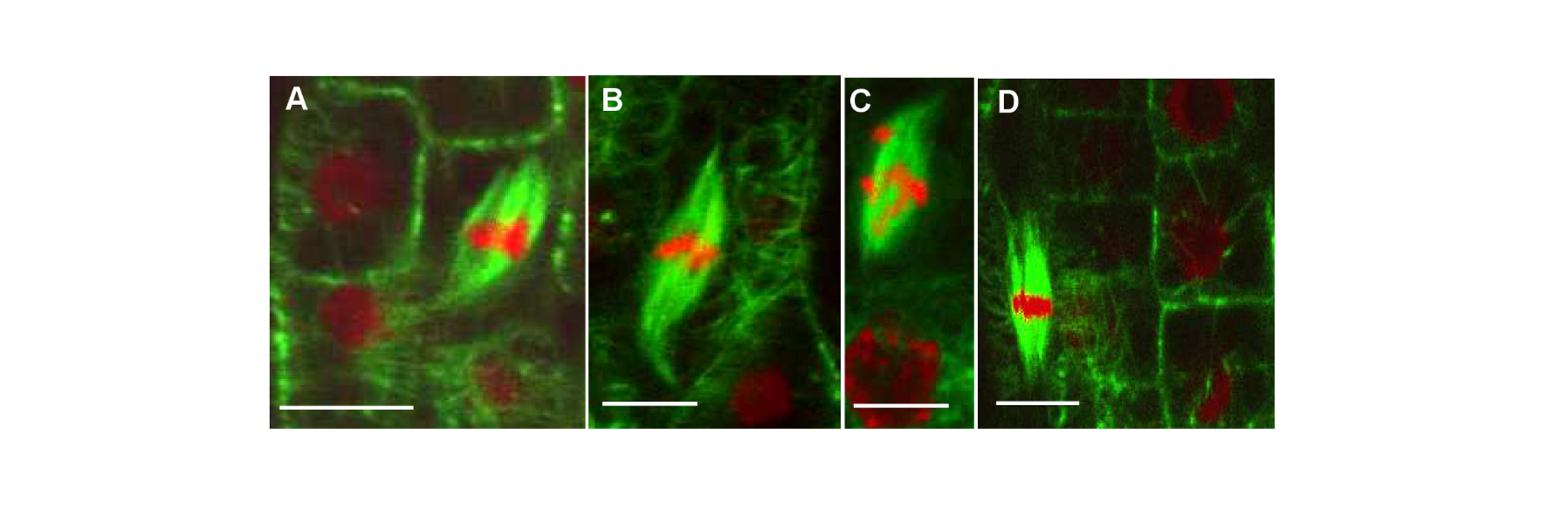

Supplement: Figure S4 — Mitotic Cell in Roots Cells of γ-Tubulin Mutants of A. thaliana Exhibit Curved Spindles and Misaligned Chromosomes. Projections of serial confocal optical sections of mutant lines co-expressing the microtubule binding domain MBD::GFP (green) and nuclear histone H2B::YFP (red) proteins. (A) Spindle in root cells of tubg1-1; (B, C) of tubg2-1 and (D) in wild-type. Bars = 5 µm. (TIF) [file ppat.1002343.s004.tif]

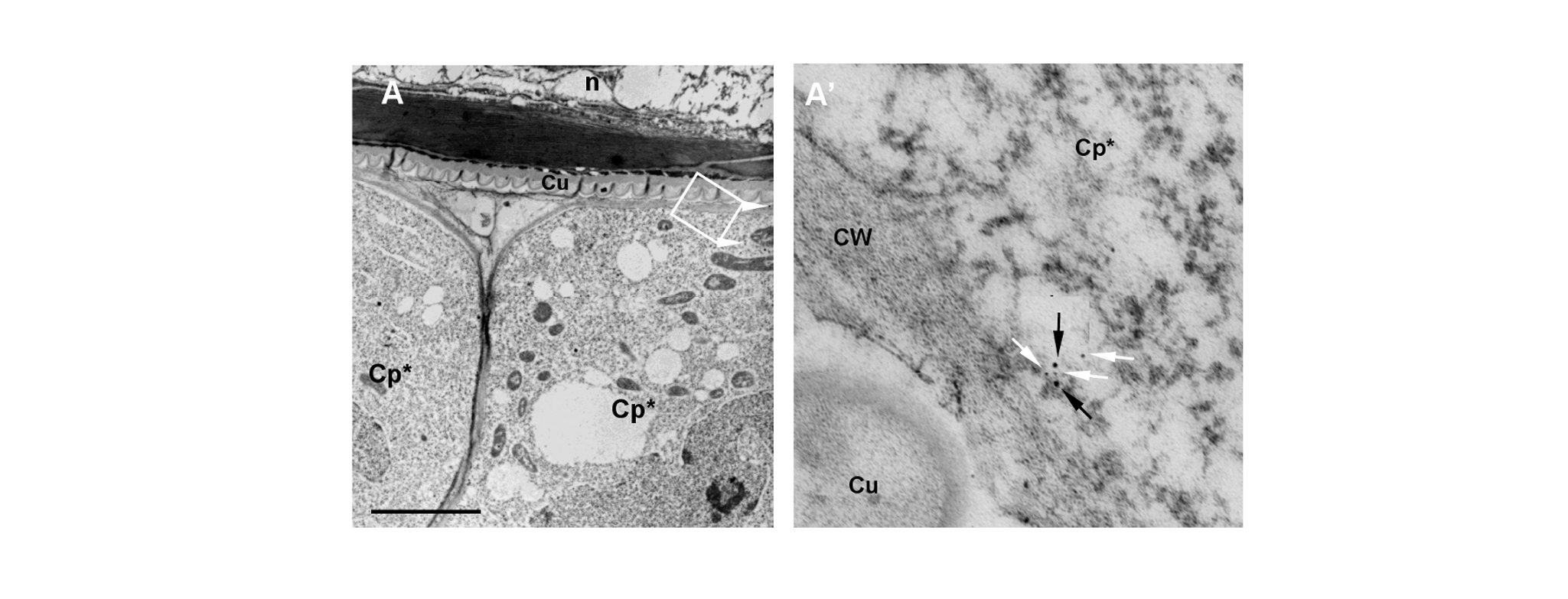

Supplement: Figure S5 — Double Immunoelectron Microscopy Localization of GCP3 and γ-Tubulin in Giant Cells. Dissected wild type galls (14 DAI) were sectioned and processed for double immunoelectron microscopy with anti-GCP3 and γ-tubulin primary antibodies, followed by secondary 10 and 5 nm gold-conjugated antibody respectively. (A and A') GCP3 co-localizes with γ-tubulin to the cell cortex. n, nematode; Cp*, giant cell cytoplasm; Cu, nematode cuticle; CW, cell wall. Bars = 300 nm (A). (TIF) [file ppat.1002343.s005.tif]

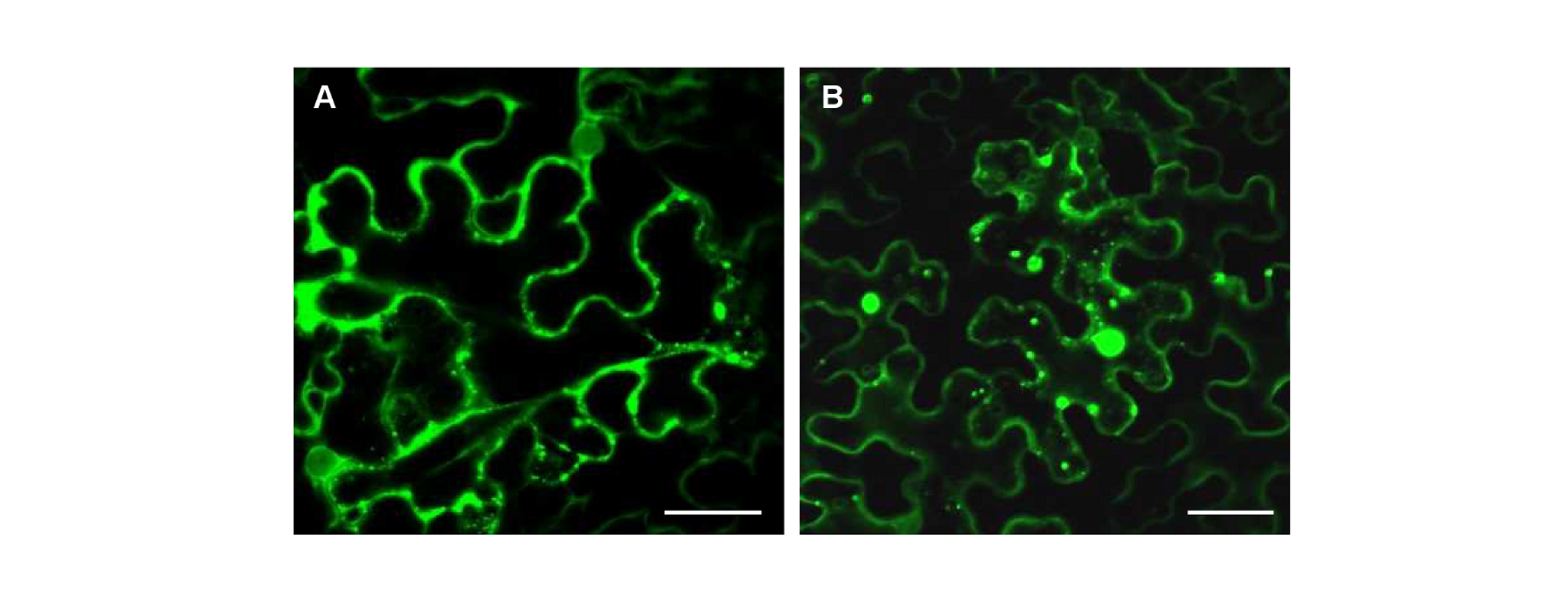

Supplement: Figure S6 — Localization of Transiently Expressed TUBG1-GFP in Leaf Pavement Cells Nicotiana tobaccum. (A) C-terminal fusion, TUBG1-GFP; and (B) N-terminal fusion GFP-TUBG1. Bar = 20 µm. (TIF) [file ppat.1002343.s006.tif]

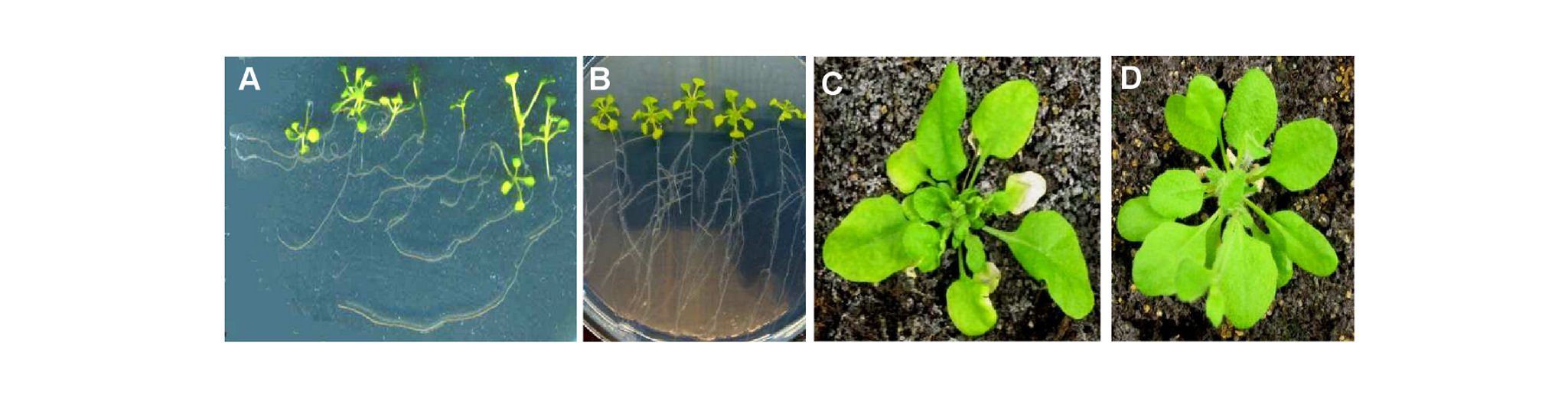

Supplement: Figure S7 — Ectopic Expression of γ-Tubulin in Arabidopsis thaliana Seedlings Causes Skewed Roots and Curled Leaves. Roots of γ-tubulin overexpressing seedlings skew to the left side (A) compared to wild type (B); γ-tubulin overexpressing seedlings exhibit leaf curling (C) compared with the wild-type (D). (TIF) [file ppat.1002343.s007.tif]

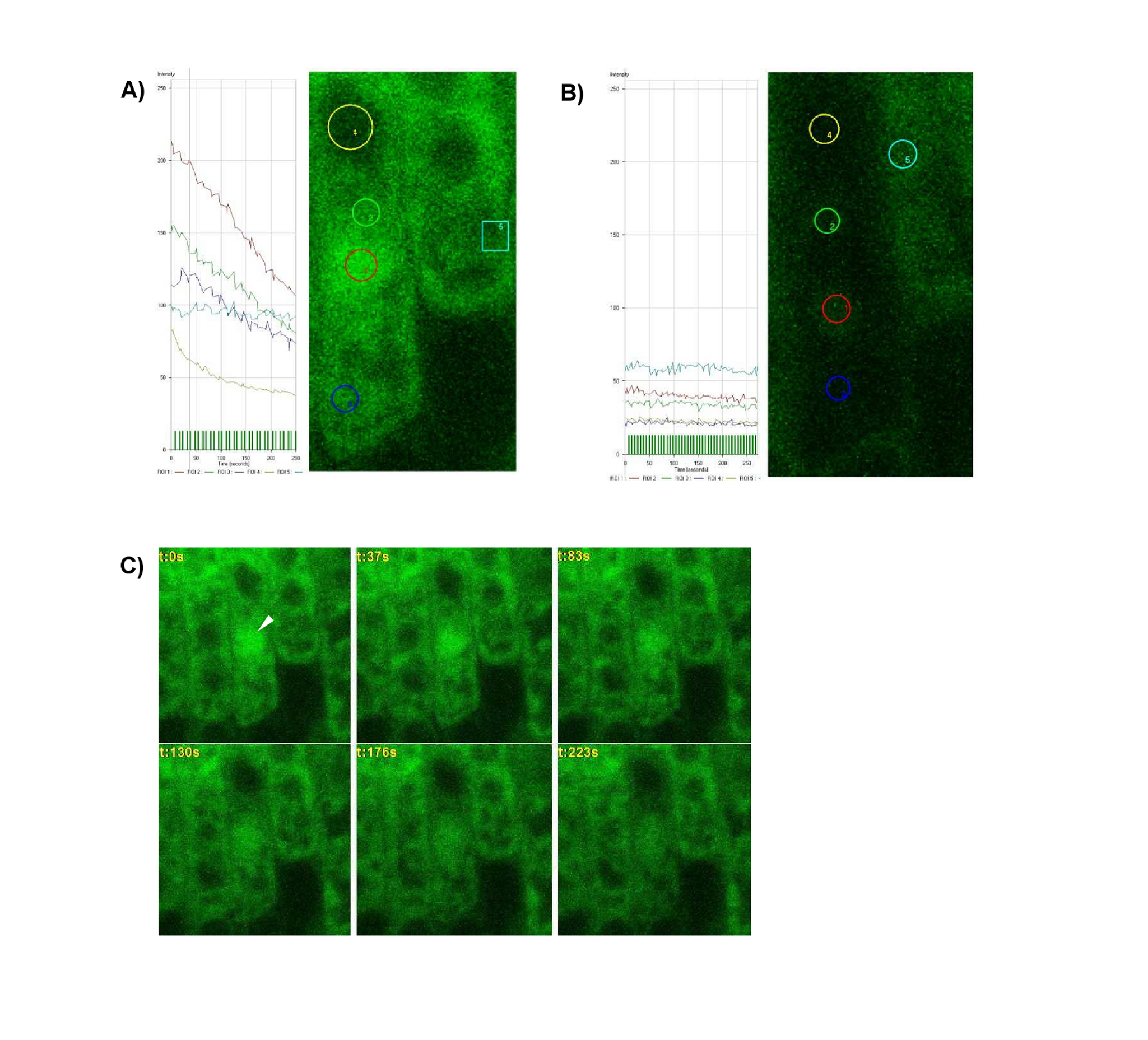

Supplement: Figure S8 — FLIP Analysis of γ-Tubulin-GFP on a Root Apical Meristem Cell of Arabidopsis thaliana. (A) Beginning of the photobleach: region of interest 1 (ROI 1; red) shows γ-tubulin-GFP in the mitotic spindle; ROI 2 (green) is a cytoplasmic region proximal to the bleach region ROI 4 (yellow). ROI 3 (violet) is a cytoplasmic region distant from the bleach region. ROI 5 (blue) is a neighbouring cell used as control. (B) Late stages of the photobleach show bleaching of the majority of GFP-γ-tubulin molecules (see graph to the left). ROI 1 (red) corresponds to the spindle position with remaining fluorescence above the background of ROI 3 (violet) and ROI 2 (green), suggesting a fraction of Sγ-tubulin bound to the spindle has a slow turnover. (C) Image representing 6 time points of the fluorescence intensity distribution in meristematic root cells following a FLIP regime of 16 bleach repeats of each 10secs. Arrowhead points to a spindle. (TIF) [file ppat.1002343.s008.tif]
