# Supplementary material for: Feeding Cells Induced by Phytoparasitic Nematodes Require γ-Tubulin Ring Complex for Microtubule Reorganization
Source: PLoS Pathog. 2011 Dec 1;7(12):e1002343. doi: 10.1371/journal.ppat.1002343 (PMC3228788; doi:10.1371/journal.ppat.1002343)
Supplement: Table S1 — Primer Sequences Used in qRT-PCR Amplification of the AtTUBG1, AtTUBG2, AtGCP3, AtGCP4, AtOXA1 and AtUBP22 Gene Transcripts. (DOCX) [file ppat.1002343.s009.docx]

**Table S1.** Primer Sequences Used in qRT-PCR Amplification of the *AtTUBG1*, *AtTUBG2, AtGCP3*, *AtGCP4*, *AtOXA1*  and *AtUBP22* Gene Transcripts.

*AtTUBG1* At3g61650 L- AAACCAGAGTAAGTCGCATGAA

R- TGCTCCTACCAAAACCCATT

*AtTUBG2* At5g05620 L- CAGGAGTTGCGGATCCTAAG

R- AATGCCAACACTCTTTATCGTT

*AtGCP3* At5g06680 L- TGGGTGAACAGTCACAGACC

R- CTCCAGCACGCTGTGTTAAG

*AtGCP4* At3g53760 L- GTGGCGTGTTAGAGAGCACA

R- TTCCAGCAGAACTGGAGACA

*AtOXA1a* At5g62050 TACCTGATCTGCCTCCACCT

*AtOXA1b* At5g62050 AACAGGACTCAGCGATGTTG

*AtUBP22* At5g10790 L- GCCAAAGCTGTGGAGAAAAG

R- TGTTTAGGCGGAACGGATAC
